# Sirtuin 4 (Sirt4) downregulation contributes to chondrocyte senescence and osteoarthritis via mediating mitochondrial dysfunction

**DOI:** 10.7150/ijbs.85585

**Published:** 2024-01-27

**Authors:** Shiyuan Lin, Biao Wu, Xinjia Hu, Huading Lu

**Affiliations:** 1 Department of Orthopaedics, The Fifth Affiliated Hospital of Sun Yat-Sen University, Zhuhai 519000, Guangdong, China.; 2 Department of Trauma Orthopedic, Shenzhen People's Hospital, The Second Clinical Medical College of Jinan University and The First Affiliated Hospital of Southern University of Science and Technology, Shenzhen 518035, Guangdong, China.

**Keywords:** Osteoarthritis, Sirt4, Mitochondria, Senescence, Reactive oxygen species

## Abstract

Chondrocyte senescence has recently been proposed as a key pathogenic mechanism in the etiology of osteoarthritis (OA). Nevertheless, the precise molecular mechanisms underlying chondrocyte senescence remain poorly understood. To address this knowledge gap, we conducted an investigation into the involvement of Sirtuin 4 (Sirt4) in chondrocyte senescence. Our experimental findings revealed a downregulation of Sirt4 expression in TBHP-induced senescent chondrocytes in vitro, as well as in mouse OA cartilage. Additionally, we observed that the knockdown of Sirt4 in chondrocytes promoted cellular senescence and cartilage degradation, while the overexpression of Sirt4 protected the cells against TBHP-mediated senescence of chondrocytes and cartilage degradation. Moreover, our findings revealed elevated levels of reactive oxygen species (ROS), abnormal mitochondrial morphology, compromised mitochondrial membrane potential, and reduced ATP production in Sirt4 knockdown chondrocytes, indicative of mitochondrial dysfunction. Conversely, Sirt4 overexpression successfully mitigated TBHP-induced mitochondrial dysfunction. Further analysis revealed that Sirt4 downregulation impaired the cellular capacity to eliminate damaged mitochondria by inhibiting Pink1 in chondrocytes, thereby enhancing the accumulation of ROS and facilitating chondrocyte senescence. Notably, the overexpression of Pink1 counteracted the effects of Sirt4 knockdown on mitochondrial dysfunction. Importantly, our study demonstrated the promise of gene therapy employing a lentiviral vector encoding mouse Sirt4, as it successfully preserved the integrity of articular cartilage in mouse models of OA. In conclusion, our findings provide compelling evidence that the overexpression of Sirt4 enhances mitophagy, restores mitochondrial function, and protects against chondrocyte senescence, thereby offering a novel therapeutic target and potential strategy for the treatment of OA.

## Introduction

Osteoarthritis (OA) is a prevalent degenerative joint disorder that poses significant threats to the well-being of affected individuals and imposes a substantial medical and socioeconomic burden on a global scale^1^,^2^. Clinically, OA manifests as joint pain, swelling, stiffness, and mobility impairment^3^. Histologically, OA is characterized by the degradation of articular cartilage, the formation of osteophytes, subchondral sclerosis, and synovial inflammation^4^. In China, over 8% of the population suffers from symptomatic OA, with an incidence rate of 62.2% in individuals aged 60 and above, and 80% in those aged 75 and above^5^. The United States is expected to witness a rise in the number of OA patients from 30 million to 67 million by 2030, with more than half of the affected individuals being 65 years or older^6^. As there is currently a lack of disease-modifying therapies, OA patients typically resort to joint replacement surgery in the advanced stages of the disease after enduring decades of joint pain and stiffness^7^. Consequently, there is an urgent need to enhance our understanding of the pathogenesis of OA to facilitate the development of novel therapeutic strategies.

Cellular senescence represents a state of permanent cell-cycle arrest triggered by cellular stress^8^. Increasing evidence supports its role in age-related functional decline, especially in musculoskeletal diseases like OA^9^. Studies show a significant accumulation of senescent chondrocytes in human OA cartilage compared to healthy cartilage, with research by Gao et al. revealing a strong correlation between chondrocyte senescence and the severity of knee articular lesions^10^-^12^. Additionally, the transplantation of senescent fibroblasts into mouse knee joints induced cartilage erosion, osteophyte formation, and loss of mobility, thereby suggesting the ability of senescent cells to modify the synovial microenvironment and induce OA-like pathology^13^. Conversely, local clearance of senescent chondrocytes attenuated the development of post-traumatic OA and fostered a pro-regenerative environment in mice^14^. Despite being in cell-cycle arrest, senescent cells exhibit metabolic activity and actively secrete pro-inflammatory cytokines, chemokines, growth factors, and extracellular matrix (ECM)-degrading proteins, collectively known as the senescence-associated secretory phenotype (SASP)^15^. The SASP factors facilitate communication between senescent chondrocytes and neighboring cells, promoting senescence in the latter^16^. While chondrocyte senescence plays essential roles in OA development, the factors that stimulate chondrocyte senescence and the underlying mechanisms remain to be identified^11^.

Recent findings emphasize the significance of mitochondrial dysfunction as a defining hallmark of cellular senescence and age-associated diseases^17^. Therefore, the preservation of mitochondrial homeostasis has become an imperative focus. Mitochondrial quality control (MQC) is the key mechanism to coordinate various mitochondrial biofunctions and preserve mitochondrial homeostasis, primarily through mitochondrial biogenesis, dynamics, autophagy and mitophagy^18^. Mitophagy, a specialized form of autophagy that selectively eliminates depolarized and dysfunctional mitochondria, emerges as a central mechanism governing mitochondrial homeostasis and sustaining energy balance^19^. Notably, impaired autophagy has been observed in senescent fibroblast-like synoviocytes and chondrocytes from patients with OA^20^,^21^. Moreover, general autophagy induction has demonstrated efficacy in mitigating the severity of experimental OA and safeguarding chondrocytes from oxidative stress^22^. Genetic studies employing the silencing of Parkin, a mitophagy regulator, in chondrocytes have unequivocally affirmed the indispensability of mitophagy in maintaining mitochondrial quality in OA^23^. Consequently, targeting mitophagy emerges as a pertinent therapeutic strategy warranting exploration for the treatment of OA.

Sirtuins, a family of NAD^+^-dependent deacylases and ADP-ribosyl-transferases, participate in a myriad of intricate cellular processes^24^. To date, seven homologs, namely SIRT1 to SIRT7, have been identified. Sirtuins exhibit distinct tissue and sub-cellular localizations, with SIRT2 being expressed in both the nucleus and cytoplasm. Conversely, SIRT3, SIRT4, and SIRT5 reside within mitochondria, while SIRT1, SIRT6, and SIRT7 localize to the nucleus. Among these homologs, Sirt4 plays pivotal roles in several biological processes, including energy metabolism, oxidative stress, tumor development, and cellular senescence^25^-^27^. Yi et al. have illuminated that the upregulation of SIRT4 in keratinocytes alleviates the senescence phenotype^28^. Furthermore, Liang et al. have substantiated that the downregulation of SIRT4 exacerbates mitochondrial damage in renal tubular epithelial cells^29^. Additionally, Dai et al. have unveiled the suppressive effect of SIRT4 on the inflammatory response in OA^30^. Despite this finding, the role of Sirt4 in mitochondrial homeostasis and cellular senescence in chondrocytes remains unclear.

In this study, we provide compelling evidence showcasing the downregulation of mitochondrial Sirt4 in both senescent chondrocytes and mouse cartilage affected by OA. Furthermore, we demonstrate that knockdown of Sirt4 in chondrocytes induces mitochondrial dysfunction and cellular senescence. Our subsequent in vivo and in vitro experiments shed light on the involvement of Sirt4 in mitophagy, unveiling novel insights into the underlying mechanism.

## Methods

### Isolation and culture of primary murine articular chondrocytes

The isolation of primary murine articular chondrocytes followed an established protocol as described previously^31^. Briefly, knee articular cartilage tissue obtained from C57BL/6 mice aged 5-10 days postpartum was collected and fragmented. The cartilage fragments underwent incubation in a collagenase D solution (3 mg/ml) at 37 ℃ with 5% CO_2_ and 95% air for 60 minutes, followed by further digestion using a collagenase D solution (0.5 mg/ml) for a duration of 12 hours. The resulting chondrocytes were then cultured in DMEM/F12 medium (Gibco, USA) supplemented with 10% fetal bovine serum (Biological Industries, USA) and 1% penicillin/streptomycin (Gibco, USA). For the present study, chondrocytes within the fourth passage were selected for experimentation. Approval for this research was obtained from the Institutional Animal Care and Use Committee (IACUC) of the Fifth Affiliated Hospital of Sun Yat-Sen University.

### Total RNA extraction and real-time quantitative polymerase chain reaction (RT-qPCR) analysis

Total RNA isolation was carried out using the Total RNA Isolation Kit (Omega Bio-Tek, USA) in strict accordance with the manufacturer's instructions. Subsequently, cDNA synthesis was performed utilizing the RevertAid First Strand cDNA Synthesis Kit (Thermo Fisher, USA), following the manufacturer's recommended protocols. RT-qPCR analysis was conducted employing the Taq Pro Universal SYBR qPCR Master Mix (Vazyme, China) on the CFX96™ Real-Time PCR Detection Systems (Bio-Rad, USA). Primer sequences employed in the amplification process can be found in Supplementary Table 1. The expression level was determined based on the threshold cycle (Ct), and relative expression levels were calculated using the 2^-ΔΔCT^ method, with β-actin serving as the reference gene.

### Protein extraction and Western blotting analysis

The extraction of total protein from chondrocytes was performed using RIPA lysis buffer (Solarbio, China) supplemented with 1% PMSF protease inhibitor (Solarbio, China). Western blotting was performed following established protocols as described in our previous work^32^. Firstly, the protein concentrations were determined using the Enhanced Bicinchoninic Acid (BCA) protein assay kit (Beyotime, China). Secondly, separation of protein samples was achieved through 10% SDS-PAGE gels (Epizyme, China), with a Prestained Protein Ladder (Thermo Scientific, USA) serving as a molecular weight marker. Subsequently, the separated proteins were transferred onto polyvinylidene difluoride (PVDF) membranes (Bio-Rad, USA). The membranes were incubated overnight at 4°C on a shaker with primary antibodies including anti-Sirt4 (1:1000, ABclonal, China), anti-Mmp13 (1:1000, Abcam, UK), anti-Col2a1 (1:1000, Abcam, UK), anti-p21 (1:1000, ABclonal, China), anti-p16 (1:1000; Abcam, UK), anti-p62 (1:1000; Abcam, UK), anti-LC3 (1:1000, Cell Signaling Technology, USA), Pink1 (1:1000; Santa Cruz Biotechnology, USA), and β-actin (1:5000; Proteintech, USA). Following incubation, membranes were washed with Tris-buffered saline containing 0.1% Tween-20 (TBST) and subsequently incubated with specific secondary antibodies at room temperature for 1 hour. After another round of TBST washing, the signals were detected using the iBright™ FL1500 Imaging System (Thermo Fisher Scientific, USA). Protein expression levels were quantified by analyzing the densitometry of the bands using ImageJ software and normalized to β-actin.

### RNA interference and gene overexpression

Small interference RNA (siRNA) specifically targeting Sirt4 (siSirt4) were purchased from Ribobio (Shanghai, China), alongside a matched negative control (siCtrl). Detailed information regarding the siRNAs employed in this investigation can be found in Supplementary Table 2. To initiate Sirt4 knockdown, cells were seeded in 6-well plates to attain 50-70% confluent growth, followed by transfection with 20nM of each siRNA using siRNA-Mate (GenePharma, China), according to the manufacturer's instructions.

Recombinant plasmids for Sirt4 overexpression (oe-Sirt4), Pink1 overexpression (oe-Pink1), and the corresponding negative control plasmid (oe-Ctrl) were constructed by Shanghai Genechem Co.Ltd Biotech (Shanghai, China). Chondrocytes were cultured in a 6-well plate with DMEM/F12 medium supplemented with 10% fetal bovine serum (FBS) until reaching 80-90% confluency. The plasmids were transfected into chondrocytes at a final concentration of 1.6 μg/ml using Lipofectamine 2000 (Thermo Fisher, USA), according to the manufacturer's instructions. Following a 5-hour transfection period, the medium was substituted with fresh medium.

The efficacy of knockdown or overexpression was assessed 48 hours post-transfection through RT-qPCR and western blotting. Once successful knockdown or overexpression was confirmed, chondrocytes were harvested for subsequent experiments.

### γ-H2AX immunofluorescence staining

Chondrocytes were fixed in 4% paraformaldehyde (PFA) for 15 minutes, followed by permeabilization with 0.1% Triton X-100 in phosphate-buffered saline (PBS) for 10 minutes. Subsequently, blocking step was carried out using 10% goat serum in PBS for 1 hour. The cells were then incubated with primary antibodies targeting γ-H2AX (1:200, Abcam, UK). After three washes with PBS, the cells were incubated with a secondary antibody, anti-rat Alexa Fluor 488 (1:500, Thermo Fisher, USA), at room temperature in the absence of light for 60 minutes. Nuclei were counterstained with DAPI (1:1000, Sigma Aldrich, USA). Finally, the stained cells were observed and imaged using a laser confocal scanning microscope (Zeiss, Germany).

### Measurement of intracellular and mitochondrial ROS

The level of mitochondrial ROS was measured by MitoSOX Red (Thermo Scientific, USA), as follows. Primary mouse chondrocytes were incubated with MitoSOX (5 μM) for 20 min at 37°C while protected from light. Cells were washed once with PBS, resuspended in PBS, and analyzed by laser confocal scanning microscope (Zeiss, Germany). The intracellular level of ROS was measured with DHE and H_2_DCFDA staining. For DHE staining, 1 μM of DHE (Thermo Scientific, USA) was employed to stain primary mouse chondrocytes, which were diluted in DMEM/F12 without sodium pyruvate and incubated for 15 minutes. As for CM-H_2_DCFDA staining, chondrocytes were subjected to staining using 5 μM of CM-H_2_DCFDA (Thermo Scientific, USA) in culture media at 37°C for 30 minutes. Subsequently, the stained cells underwent a triple wash with PBS, followed by fixation in a 4% PFA solution for 15 minutes and mounting in PBS. The samples were assayed by flow cytometry at 405 nm (Beckman, USA) or laser confocal scanning microscope (Zeiss, Germany).

### EdU assay

To assess chondrocyte proliferation, we conducted EdU staining following the manufacturer's protocol utilizing the BeyoClick™ EdU Cell Proliferation Kit with Alexa Fluor 555 (Beyotime Biotechnology, China). In brief, the cells were subjected to a 2-hour incubation at 37 °C subsequent to the addition of EdU to the culture media, resulting in a final concentration of 10 μM. Following this, the cells were fixed for 15 minutes using 4% paraformaldehyde (PFA) and permeabilized for 10 minutes using 0.2% Triton X-100. Subsequently, the cells underwent staining in the absence of light for 30 minutes with the click reaction solution, followed by counterstaining with Hoechst 33342. To determine the number of EdU positive cells, fluorescence microscopy (Olympus, Japan) was employed, and the percentage of EdU positive cells was quantified and reported.

### Cell Counting Kit‑8 (CCK‑8) assay

For the assessment of chondrocyte proliferation, cells were seeded in quintuplicate in 96-well plates at a density of 2,000 cells per well. Subsequently, they were incubated with DMEM/F12 containing 10% FBS for the respective duration. Each well was supplemented with 100 μl of culture medium. Following the incubation period, a 10 μl solution of CCK-8 (Beyotime Biotechnology, China) was added to each well and incubated for an additional 3 hours at 37 °C. Finally, the optical density (OD) values at 450 nm were measured using a luminometer (BioTek uQuant, USA).

### Senescence-associated β-galactosidase (SA-β-Gal) staining

The SA-β-gal activity in chondrocytes was assessed using the SA-β-gal staining kit (Solarbio, China) following the manufacturer's guidelines. In brief, the cells underwent three washes with PBS and were subsequently fixed with a fixative solution for a duration of 15 minutes. Following fixation, the cells were washed again and incubated in the SA-β-Gal staining solution at 37 °C for 14 hours. The proportion of SA-β-Gal-positive cells was determined and analyzed within five randomly chosen fields per biological replicate using an optical microscope (Olympus, Japan).

### Mitochondrial morphology assessment

Chondrocytes were subjected to incubation with 250 nM MitoTracker Deep Red (Invitrogen, USA) for a period of 20 minutes. Subsequently, images were acquired from randomly chosen fields of view utilizing a confocal microscope (Zeiss, Germany). Assessment and analysis of mitochondrial morphology were performed using the semiautomated morphometric tool MiNA within Fiji software.

### Mitochondrial Mass Analysis

The fluorescent probe 10-N-nonyl-acridine orange (NAO) is widely used to evaluate mitochondrial mass^33^. Briefly, cells were subjected to a 20-minute incubation with NAO (Thermo Fisher, USA) at 37°C. Following the incubation period, the cells underwent two washes with PBS and were subsequently analyzed using flow cytometry (Beckman, USA) at an excitation wavelength of 495 nm and an emission wavelength of 519 nm.

### Detection of the co-localization of mitochondria with lysosomes

Mitophagy was assessed based on the fluorescence co-localization of mitochondria with lysosomes. Specifically, MitoTracker (Thermo Fisher, USA) and LysoTracker (Beyotime Biotechnology, China) were utilized to label mitochondria and lysosomes, respectively. Live chondrocytes were co-incubated with MitoTracker Red and LysoTracker Green for a duration of 30 minutes, with concentrations determined according to the manufacturer's guidelines. Images were acquired using a laser confocal microscope (Zeiss, Germany).

### Mitochondrial membrane potential (ΔΨm) assay

The mitochondrial membrane potential (ΔΨm) of chondrocytes was assessed using the JC-1 mitochondrial membrane potential assay kit (Beyotime Biotechnology, China). In brief, at 48 hours post-transfection, chondrocytes were subjected to PBS washing and subsequently incubated in fresh culture medium. Then, chondrocytes were incubated with JC-1 in a 37 °C incubator for a duration of 20 minutes. After the incubation period, chondrocytes were washed twice with prechilled buffer and then covered with fresh culture medium. Subsequently, the cells were analyzed using laser confocal microscopy (Zeiss, Germany).

### Adenosine triphosphate (ATP) concentration assay

The intracellular ATP content was quantified using an Enhanced ATP assay kit (Beyotime Biotechnology, China) in accordance with the manufacturer's protocols. Briefly, the cells were lysed using lysis buffer and then centrifuged at 12,000 g for 5 minutes at 4 °C to obtain the supernatants for ATP detection. To perform the assay, 50 μl of the supernatant was promptly mixed with 100 μl of dilution buffer containing preincubated luciferase at room temperature for 3 minutes. Luminance (RLU) was measured using an EnVision 2105 Multimode Plate reader (PerkinElmer, USA). The concentration of ATP was determined based on a standard curve and subsequently normalized using the cellular protein level.

### Transmission electron microscopy (TEM) analysis

The chondrocytes were harvested and subsequently fixed with 2.5% glutaraldehyde for a duration of 30 minutes at room temperature. Following fixation, the chondrocytes underwent dehydration through a series of graded ethanol and acetone concentrations. Subsequently, they were embedded in the Epon mixture and sectioned into ultrathin slices. Finally, the images were acquired utilizing a transmission electron microscope (Hitachi, Japan).

### Animals and experimental OA induced by DMM

All animal procedures were conducted in accordance with the guidelines approved by the Institutional Animal Care and Use Committee (IACUC) protocol of the Fifth Affiliated Hospital of Sun Yat-Sen University (Approval number: IACUC-00318). C57BL/6 male mice used in this study were procured from the Guangdong Provincial Medical Laboratory Animal Center and housed under standard animal housing conditions. The experimental OA model was induced in 12-week-old male mice through destabilization of the medial meniscus (DMM) surgery, following established protocols^34^. Briefly, OA was induced in the right knee joint by dissecting the medial meniscotibial ligament. For the sham group, only the skin of the right knee joint was resected without inducing OA. The mice were randomly allocated into three groups (n=6 per group): the sham group, the DMM + Mock group (control lentivirus), and the DMM + oe-Sirt4 group (lentivirus encoding Sirt4). A total of 7 μl of solution, containing oe-Sirt4 or the control vector virus (approximately 10^10^ virus particles), was slowly injected into the right articular cavity using a 33-G needle with a microliter syringe. Lentivirus injection was performed at 1- and 4-weeks post DMM surgery. At 8 weeks post-surgery, the mice were euthanized, and the right knee joints were harvested for subsequent histological assessment.

### Histological section

After an interval of eight weeks post-surgery, the mice were euthanized, and the right knee joint samples were fixed using 4% PFA for a duration of 24 hours. Subsequently, the knee joint samples were subjected to decalcification in a 10% ethylenediaminetetraacetic acid (EDTA) buffered saline solution (Solarbio, China, pH = 7.2). The decalcification process involved changing the solution every 3 days, and it was completed within 4 weeks. After decalcification, the samples were embedded in Optimal Cutting Temperature compound (OCT). The knee joint samples were then sectioned in a sagittal orientation with a thickness of 10 μm, targeting the medial compartment loading area, using -20°C freezing microtomes (Thermo Fisher Scientific, USA) for subsequent histological analysis.

### Hematoxylin and eosin (HE) staining

HE staining were performed using the HE staining Kit (Beyotime, China) following the manufacturer's instructions. Briefly, the frozen sections were gently rinsed in distilled water and subsequently stained with hematoxylin solution for 5 minutes. Afterward, the sections were washed in running tap water for 10 minutes. The differentiation process was carried out in 1% acid alcohol for 10 seconds, followed by another wash in running tap water for 10 minutes. Subsequently, counterstaining was achieved by immersing the sections in eosin solution for 30 seconds, followed by a 5-minute wash in running tap water. To complete the staining process, the sections were dehydrated and cleared using 95% ethyl alcohol, absolute ethyl alcohol, and xylene, each performed twice for 2 minutes. Finally, the sections were sealed using the resinous medium.

### Safranin O and Fast Green Staining

Safranin O and Fast Green staining were performed using the Modified Safranine-O and Fast Green Stain Kit (Solarbio, China) in accordance with the manufacturer's protocols. Briefly, the frozen sections were hydrated with distilled water and subsequently stained with Weigert's Iron Hematoxylin for 5 minutes. Following this, gentle washing with distilled water was performed until no excess dye leached out from the sections, and differentiation was carried out using 1% Acid-Alcohol for 15 seconds. The sections were then rinsed gently with distilled water and stained with 0.2% Fast Green for 5 minutes. Quick rinsing with a 1% acetic acid solution for 10 seconds was conducted, and the sections were subsequently stained with 0.1% safranin O solution for 5 minutes. Dehydration and clearing steps were carried out using 95% ethyl alcohol, absolute ethyl alcohol, and xylene, each step performed twice for 2 minutes. Finally, the sections were mounted with a resinous medium. Cartilage destruction in the mouse knee joints was evaluated using the Osteoarthritis Research Society International (OARSI) guideline, which provided both photograph references and text instructions for scoring^35^.

### Immunofluorescence

The frozen sections were subjected to hydration in distilled water, followed by permeabilization with 0.2% Triton-X for a duration of 10 minutes. Gentle washing with PBS was then performed, and the sections were blocked for 1 hour at room temperature using the block solution (5% goat serum). Subsequently, the sections were incubated with the primary antibody in the block solution for 14 hours at 4°C. For visualization, the sections were stained with Alexa 488 or Alexa 568 dye-labeled secondary antibodies (1:500, Thermo Fisher, USA) for 2 hours at room temperature, with aluminum foil covering to shield from light. Then the sections were washed twice gently with PBS for 5 minutes each. To visualize nuclei, the sections were stained with DAPI solution (1:2000, Beyotime, China) for 5 minutes, followed by washing in PBS with three changes, and finally mounted using a resinous medium. Images were captured using a laser confocal microscope (Zeiss, Germany). The proportion of positive fluorescence cells in the medial tibial plateau area was determined.

### Statistical analysis

The data is presented as mean ± standard deviation (SD). For data conforming to a normal distribution, the present study employed independent Student's t-tests and one-way analysis of variance (ANOVA). Statistical significance was set at p < 0.05. Statistical analyses were conducted using SPSS 20.0 (SPSS, Inc., Chicago, IL, USA) and GraphPad Prism 8.3 (GraphPad Software Inc., La Jolla, CA, USA). All experiments were replicated independently a minimum of three times.

## Results

### Sirt4 is downregulated in OA chondrocytes and cartilage tissue affected by OA

To induce cellular senescence and OA phenotype in vitro, chondrocytes were subjected to varying concentrations of Tert-butyl hydroperoxide (TBHP)^36^. The assessment of p16 and p21 expression, conventional biomarkers of senescence, was carried out using western blotting. Due to the intimate connection between OA and ECM metabolism, an investigation into the expression levels of ECM-related proteins, namely Col2a1 and Mmp13, was conducted using western blotting analysis. Following a 48-hour TBHP treatment, there was a dose-dependent increase in the protein levels of p16, p21, and Mmp13, while the protein level of Col2a1 decreased (Supplementary Fig. 1A-E). Correspondingly, the proportion of SA-β-Gal positive chondrocytes, indicating senescence, increased in a dose-dependent manner upon TBHP treatment (Supplementary Fig. 1F and G). Additionally, chondrocytes treated with 50μM TBHP for 48 hours exhibited elevated mRNA expression of SASP genes, including Cxcl10, IL-6, Mcp1, Mmp3, IL-1β, TNFα, and IL-17, as compared to non-treated controls (Supplementary Fig. 1H-N). Notably, the expression level of Mmp10 showed no significant difference in chondrocytes treated with or without TBHP (Supplementary Fig. 1O). These results collectively indicated that chondrocytes treated with 50μM TBHP display distinct senescent phenotypes and OA-like changes in vitro. As a result, this concentration was selected for all subsequent experiments.

**Figure 1:**
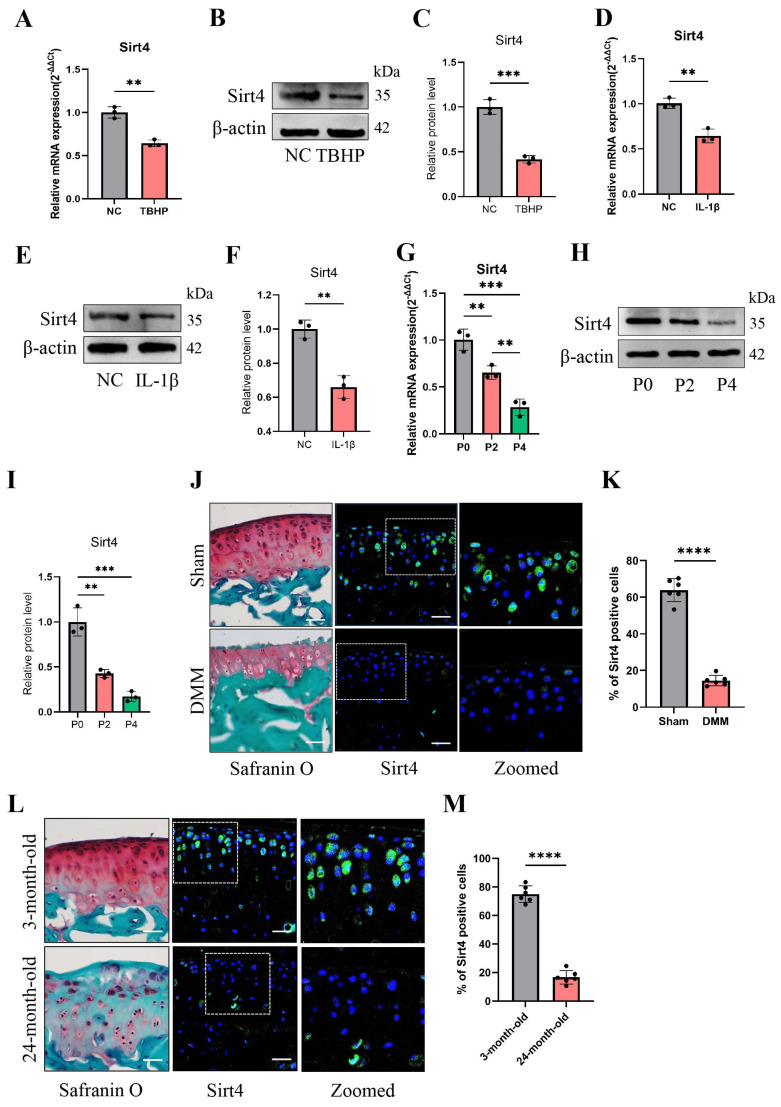
** Sirt4 is down-regulated in OA chondrocytes and cartilage tissue affected by OA.** (A-C) RT-qPCR and western blotting analysis of Sirt4 mRNA and protein expression level in primary mouse chondrocytes treated with and without 10 ng/mL IL-1β for 48h. Band intensity relative to β-actin. n = 3, **p < 0.01. (D-F) RT-qPCR and western blotting analysis of Sirt4 mRNA and protein expression level in primary mouse chondrocytes treated with and without 50 μM TBHP for 48h. Band intensity relative to β-actin. n = 3, **p < 0.01, ***p < 0.001. (G-I) RT-qPCR and western blotting analysis of Sirt4 mRNA and protein expression level during passaging of primary mouse chondrocytes. Band intensity relative to β-actin. n = 3, **p < 0.01, ***p < 0.001. (J, K) Safranin O staining of cartilage sections and immunofluorescence of Sirt4 from control (sham) and posttraumatic OA cartilage (8 weeks after DMM) of mice. The Sirt4-positive cells were quantified. n = 6, ****p < 0.0001, Scale bars: 50μm. (L, M) Safranin O staining of cartilage sections and immunofluorescence of Sirt4 from 3- and 24-month-old mice. The Sirt4-positive cells were quantified. n = 6, ****p < 0.0001, Scale bars: 50μm.

To investigate the potential correlation between Sirt4 and chondrocyte senescence in OA, an in-depth analysis of Sirt4 expression levels was performed in primary mouse chondrocytes exposed to TBHP or interleukin-1β (IL-1β). IL-1β is widely employed to simulate pathological OA conditions and establish an in vitro OA model^37^. Utilizing RT-qPCR and western blotting techniques, a significant reduction in Sirt4 mRNA and protein levels was observed in chondrocytes treated with TBHP or IL-1β compared to untreated controls (Fig. 1A-F). Notably, RT-qPCR analyses revealed a downregulation in the expression levels of Sirt3 and Sirt5, both of which are localized in the mitochondria, in chondrocytes subjected to TBHP treatment as opposed to untreated controls (Supplementary Fig. 2). Additionally, RT-qPCR and western blotting analyses revealed a substantial down-regulation of Sirt4 expression during successive passages of primary mouse chondrocytes (Fig. 1G-I). Following this, we conducted a detailed analysis of Sirt4 expression in the articular cartilage of two distinct mouse models: the destabilization of the medial meniscus (DMM) surgery-induced posttraumatic OA model and the aging-associated OA model. Consistent with earlier observations, the protein level of Sirt4 exhibited a significant decrease in the cartilage of both OA mouse models (Fig. 1J-M). These collective findings imply an inverse correlation between Sirt4 expression and chondrocyte senescence, underscoring its potential significance in OA.

### Sirt4 knockdown leads to senescence of chondrocytes and cartilage degradation

**Figure 2:**
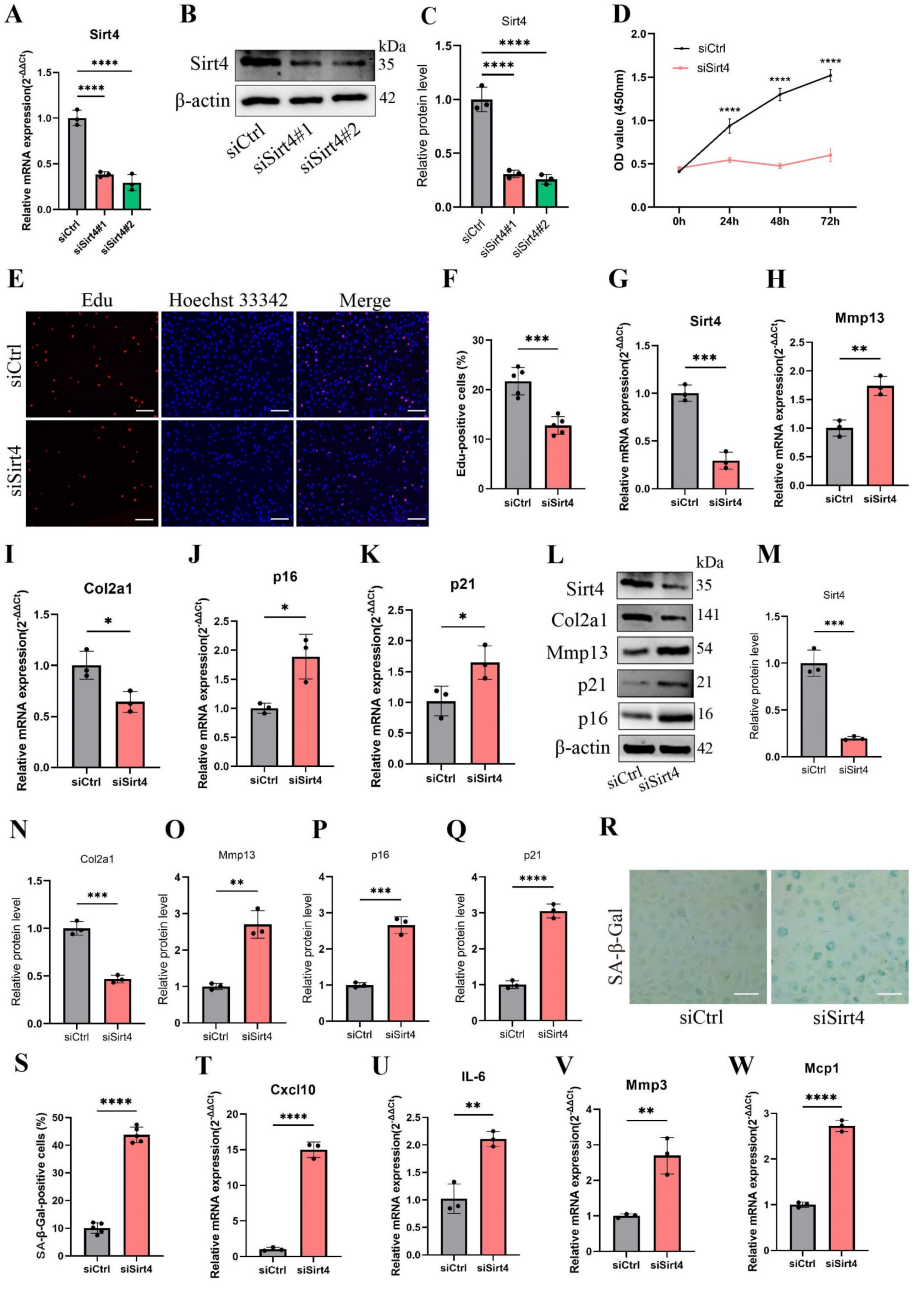
** Sirt4 knockdown induces chondrocyte senescence and cartilage degradation.** (A) Relative mRNA expression level of Sirt4 in primary mouse chondrocytes transfected with siRNA targeting Sirt4 (siSirt4) compared to negative control siRNA (siCtrl). β-actin was used as normalization control. n = 3, ****p < 0.0001. (B, C) Western blotting analysis of Sirt4 protein expression level in primary mouse chondrocytes transfected with siSirt4 compared to siCtrl. Band intensity relative to β-actin. n = 3, ****p < 0.0001. (D) CCK-8 assay was used to determinate the proliferative capacities of primary mouse chondrocytes transfected with siSirt4 compared to siCtrl. n=5, ****p < 0.0001. (E, F) Representative EdU staining images of primary mouse chondrocytes indicated a decrease in cell proliferation in Sirt4 knockdown group. DAPI staining was performed to visualize all nuclei of the cells tested. The bar graph shows quantification (%) of the EdU-positive cell population. n = 5, ****p < 0.0001. Scale bars: 100 μm. (G-K) RT-qPCR analysis was used to determinate the mRNA expression levels of Sirt4, Mmp13, Col2a1, p16 and p21 in primary mouse chondrocytes transfected with siSirt4 compared to siCtrl. β-actin was used as normalization control. n = 3, *p < 0.05, **p < 0.01, ***p < 0.001. (L-Q) Western blotting analysis was used to determinate the protein expression levels of Sirt4, Mmp13, Col2a1, p16 and p21 in primary mouse chondrocytes transfected with siSirt4 or siCtrl. Band intensity relative to β-actin. n = 3, **p < 0.01, ***p < 0.001, ****p < 0.0001. (R, S) SA-β-Gal staining and quantification of SA-β-Gal positivity in primary chondrocytes transfected with siSirt4 or siCtrl. The SA-β-gal positive cells were quantified using ImageJ. n = 5, ****p < 0.0001. Scale bars: 50 μm. (T-W) RT-qPCR analysis was used to determinate the mRNA expression levels of Cxcl10, IL-6, Mmp3 and Mcp1 in primary mouse chondrocytes transfected with siSirt4 or siCtrl. β-actin was used as normalization control. n = 3, **p < 0.01, ****p < 0.0001.

To determine the role of Sirt4 in chondrocyte senescence in vitro, we designed two small-interfering RNAs (siRNAs) to suppress the expression of Sirt4. RT-qPCR and western blotting exhibited that both of the two siRNAs have high silencing efficiency, so siSirt4#2 was randomly selected for follow-up experiments (Fig. 2A-C). Compared with chondrocytes treated with negative control siRNA (siCtrl), those treated with siSirt4 had lower OD values at 450nm for 24, 48 or 72 h, respectively (Fig. 2D). Consistently, EdU assay indicated that silence of Sirt4 inhibited the viability of chondrocytes (Fig. 2E, F). In addition, RT-qPCR and western blotting showed that downregulation of Sirt4 resulted in a significant reduction of Col2a1 expression but increase of Mmp13, p16 and p21 expression (Fig. 2G-Q), implying that Sirt4 may be a primary regulator of cartilage ECM and cellular senescence. Correspondingly, Sirt4 knockdown increased the percentage of SA-β-Gal-positive chondrocytes (Fig. 2R, S). Next, we further detected the expression of SASP-related genes by RT-qPCR. As expected, SASP-related genes, including Cxcl10, IL-6, Mcp1 and Mmp3, were significantly upregulated in Sirt4 knockdown chondrocytes as compare to the negative control group (Fig. 2T-W). Interestingly, the expression of TNFα and Mmp10 had no significant differences (Supplementary Fig. 3). Taken together, these data provide evidence that Sirt4 knockdown plays a pivotal role in promoting chondrocyte senescence, accelerating articular ECM degradation and regulating the occurrence and progression of OA.

### Sirt4 overexpression protects chondrocytes from TBHP-induced cellular senescence and cartilage degradation

**Figure 3:**
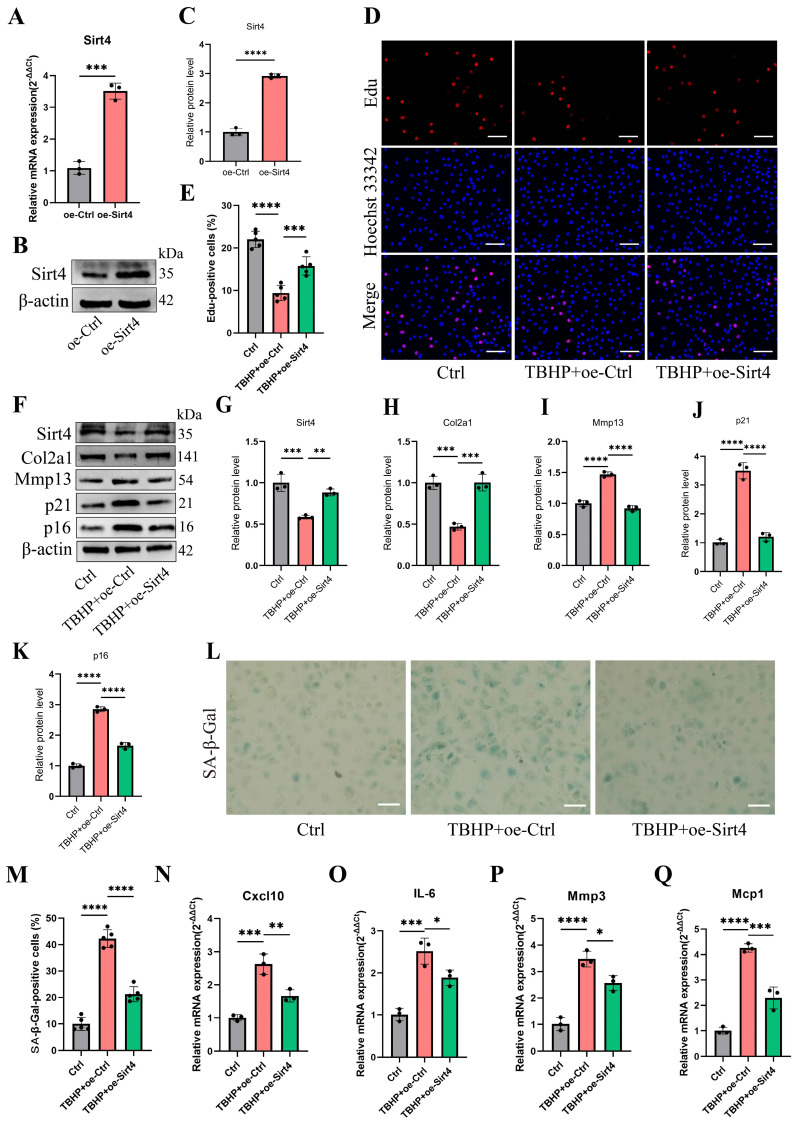
** Sirt4 overexpression attenuates TBHP-induced chondrocyte senescence and cartilage degradation.** (A) Relative mRNA expression level of Sirt4 in primary mouse chondrocytes transfected with plasmid encoding Sirt4 (oe-Sirt4) or plasmid control with no insert (oe-Ctrl). β-actin was used as normalization control. n = 3, ***p < 0.001. (B, C) Western blotting analysis of Sirt4 protein level in primary mouse chondrocytes transfected with oe-Sirt4 plasmid or oe-Ctrl plasmid. Band intensity relative to β-actin. n = 3, ****p < 0.0001 (D, E) Primary mouse chondrocytes were transfected with oe-Ctrl plasmid or oe-Sirt4 plasmid and treated or untreated with TBHP (50 μM). Representative EdU staining images of primary mouse chondrocytes indicated proliferation cells. DAPI staining was performed to visualize all nuclei of the cells tested. The bar graph shows quantification (%) of the EdU-positive cell population. n = 5, ***p < 0.001, ****p < 0.0001. Scale bars: 100 μm. (F-K) Protein levels of Sirt4, Col2a1, Mmp13 and the senescence-related markers were analyzed in primary mouse chondrocytes using western blotting. Band intensity relative to β-actin. n = 3, **p < 0.01, ***p < 0.001, ****p < 0.0001. (L, M) Representative SA-β-gal (green fluorescence) staining images of primary mouse chondrocytes indicated senescent cells. The bar graph shows quantification (%) of the SA-β-gal positive cell population. n = 5, ****p < 0.0001. Scale bars: 50 μm. (N-Q) RT-qPCR analysis was used to determinate the mRNA expression levels of Cxcl10, Il-6, Mmp3 and Mcp1 in primary mouse chondrocytes transfected with oe-Ctrl plasmid or oe-Sirt4 plasmid and treated or untreated with TBHP (50 μM). β-actin was used as normalization control. n = 3, *p < 0.05, **p < 0.01, ***p < 0.001, ****p < 0.0001.

To further investigate the effects of Sirt4 on TBHP-induced senescence, the chondrocytes were transfected with plasmid encoding Sirt4 (oe-Sirt4) or plasmid control with no insert (oe-Ctrl). The cells were subsequently cultured for 2 days with or without TBHP (50 μM). The transduction efficiency of the oe-Sirt4 plasmid was confirmed in primary mouse chondrocyte by RT-qPCR and western blotting analysis (Fig. 3A-C). Sirt4 overexpression mitigated TBHP-induced proliferation impairment in chondrocytes (Fig. 3D, E). In comparison to TBHP-treated chondrocytes, the application of the oe-Sirt4 plasmid treatment elicited augmented protein expression of Col2a1 and concomitantly decreased levels of Mmp13, p16, and p21, demonstrating that addition of Sirt4 can counteract the increase in ECM catabolism and senescence caused by TBHP (Fig. 3F-K). Consistently, Sirt4 overexpression attenuated the senescence phenotypes observed in TBHP-treated chondrocytes, as evidenced by the reduction in the proportion of SA-β-Gal-positive cells (Fig. 3L, M) and the diminished expression of SASP factors (Fig. 3N-Q). Collectively, these data suggest that Sirt4 overexpression played an essential role in protecting chondrocytes from senescence, ECM degradation and OA-like phenotypes.

### Sirt4 knockdown increases ROS production in chondrocytes

**Figure 4:**
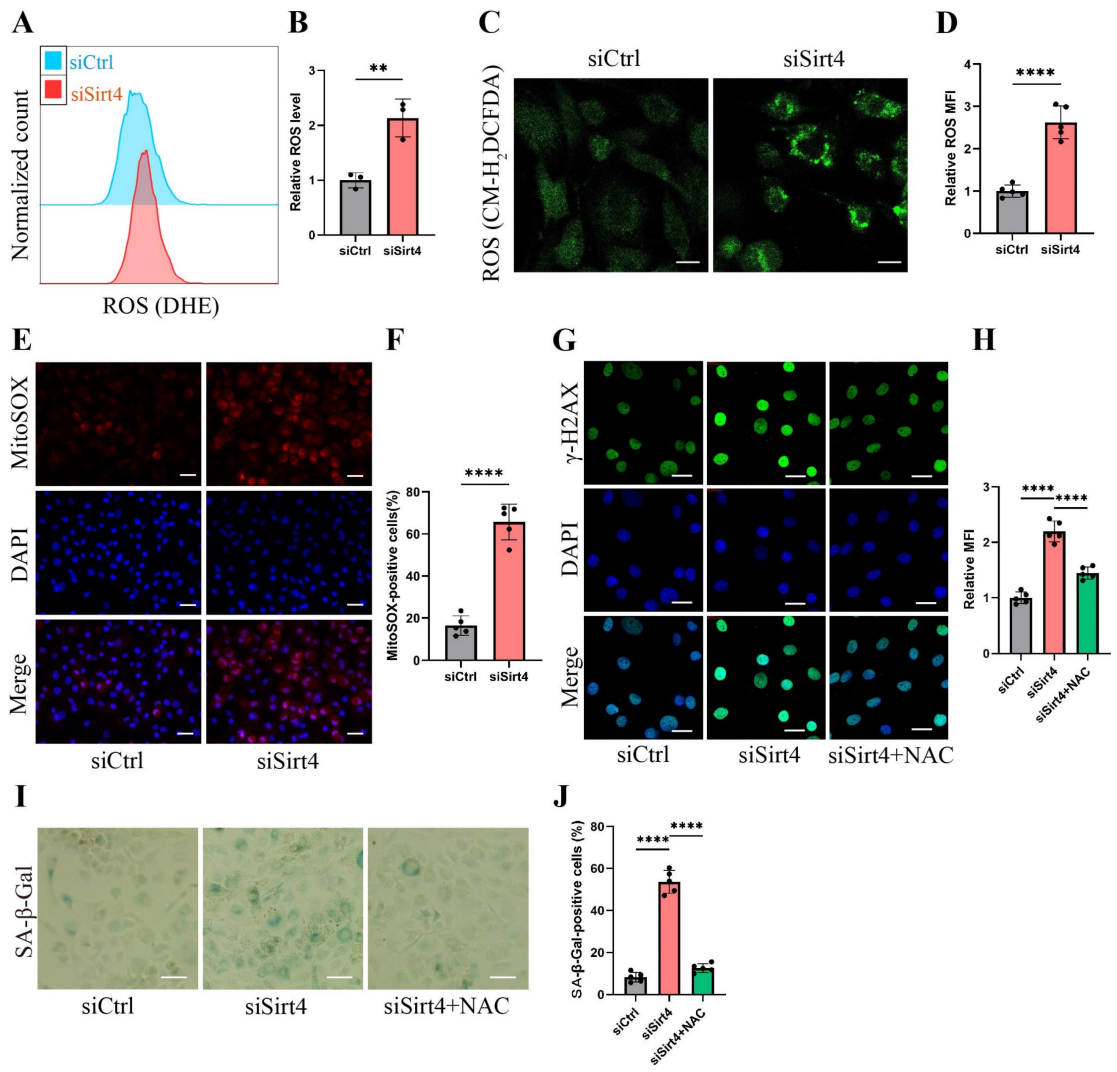
** Sirt4 knockdown increases ROS production in chondrocytes.** (A, B) Flow cytometer analysis of DHE fluorescence measuring levels of intracellular O2^•-^ in primary mouse chondrocytes transfected with siSirt4 or siCtrl. The bar graph shows the fluorescence intensity of the DHE. n = 3, **p < 0.01. (C, D) Confocal imaging analysis of CM-H_2_DCFDA fluorescence measuring levels of intracellular ROS in primary mouse chondrocytes transfected with siSirt4 or siCtrl. The bar graph shows the fluorescence intensity of the CM-H_2_DCFDA. n = 5, ****p < 0.0001, Scale bars: 20 μm. (E, F) Confocal imaging analysis of MitoSOX fluorescence measuring levels of mitochondrial ROS in primary mouse chondrocytes transfected with siSirt4 or siCtrl. The bar graph shows the fluorescence intensity of the MitoSOX. n = 5, ****p < 0.0001, Scale bars: 20 μm. (G, H) Immunofluorescence staining of γ-H2AX and fluorescence intensity of γ-H2AX in chondrocytes pretreated with 1 mM NAC for 1 h prior to transfection with siSirt4 for 48h. n = 5, ****p < 0.0001, Scale bars: 20 μm. (I, J) Representative SA-β-gal (green fluorescence) staining images of chondrocytes pretreated with 1 mM NAC for 1 h prior to transfection with siSirt4 for 48h. The bar graph shows quantification (%) of the SA-β-gal positive cell population. n = 5, ****p < 0.0001, Scale bars: 50 μm.

Mounting evidence underscores the pivotal role of ROS-triggered oxidative damage in the onset of chondrocyte senescence^38^,^39^. Thus, our study delved into the potential mediation of ROS generation in chondrocytes by Sirt4. As anticipated, the knockdown of Sirt4 yielded a marked elevation in intracellular ROS levels, as substantiated by both flow cytometric analysis and confocal imaging, utilizing two redox-sensitive fluorescent indicators, namely dihydroethidium (DHE) and CM-H_2_DCFDA that measure levels of intracellular O^2•-^and ROS, respectively (Fig. 4A-D). Moreover, the results from MitoSOX staining revealed a notable escalation in mitochondrial ROS levels in chondrocytes treated with siSirt4 compared to the negative control (Fig. 4E-F). To further elucidate the mechanistic interrelation between Sirt4 knockdown and the induction of cellular senescence, we meticulously tracked the accumulation of DNA damage subsequent to Sirt4 knockdown.

The chondrocytes subjected to Sirt4 knockdown exhibited conspicuous amplification in the DNA damage response, signified by the strong formation of γ-H2AX nuclear foci (Fig. 4G-H). We hypothesized that the accumulation of ROS caused by Sirt4 deficiency is responsible for eliciting persistent DNA damage and cellular senescence in chondrocytes. Supporting this hypothesis, chondrocytes underwent pretreatment with 5mM N-acetylcysteine (NAC) for 1 hour before transfection with siSirt4 for 48 hours. NAC, a widely recognized antioxidant with the ability to significantly impede ROS production, was chosen for its well-established efficacy^40^. The results demonstrated that NAC treatment effectively counteracted the heightened γ-H2AX nuclear foci and increased proportion of SA-β-gal-positive cells resulting from Sirt4 knockdown (Fig. 4G-J). Taken together, the aforementioned results indicate that the knockdown of Sirt4 led to heightened ROS production, subsequently triggering DNA damage and chondrocyte senescence.

### Sirt4 overexpression decreases TBHP-induced ROS production in chondrocytes

To provide additional evidence of the potential of Sirt4 overexpression in reducing ROS levels within senescent chondrocytes induced by TBHP, we implemented Sirt4 overexpression by introducing the oe-Sirt4 plasmid into chondrocytes. Subsequently, chondrocytes were subjected to treatment with TBHP (50 μM) for a duration of 48 hours. In line with expectations, both the analytical outcomes from flow cytometry and the findings obtained through confocal imaging collectively unveiled a discernible reduction in ROS levels within chondrocytes and mitochondria treated with TBHP and oe-Sirt4 plasmid, as compare to chondrocytes treated with TBHP alone. (Supplementary Fig. 4A-F). Meanwhile, the overexpression of Sirt4 counteracted the TBHP-driven escalation in the mean fluorescence intensity of γ-H2AX positive cells (Supplementary Fig. 4G, H).

### Sirt4 knockdown leads to mitochondrial dysfunction in chondrocytes

**Figure 5:**
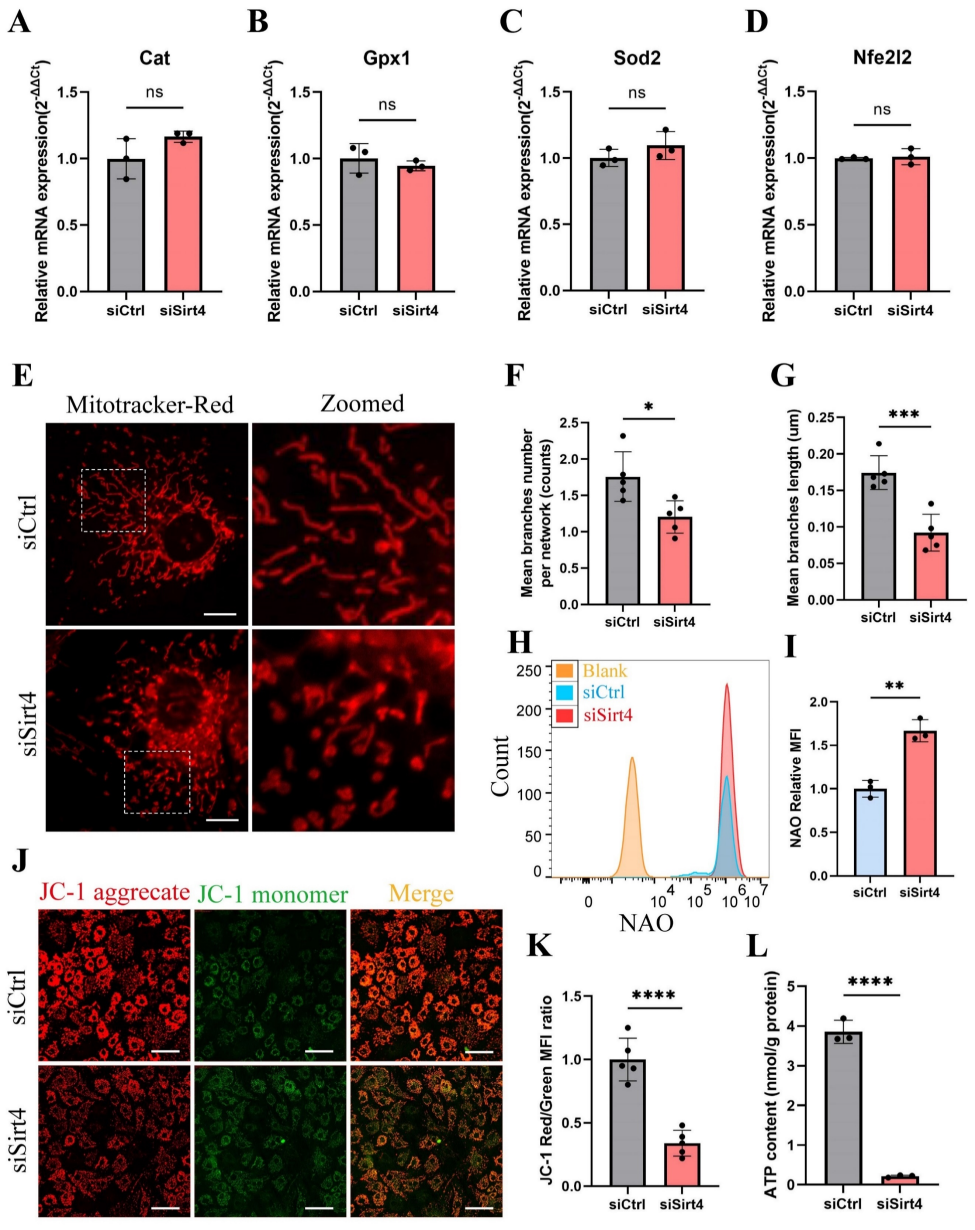
** Sirt4 knockdown leads to mitochondrial dysfunction in chondrocytes.** (A-D) RT-qPCR analysis was used to determinate the mRNA expression levels of Cat, Gpx1, Sod2 and Nfe2l2 in primary mouse chondrocytes transfected with siSirt4 or siCtrl. β-actin was used as normalization control. n = 3. (E) Mitochondrial network was analyzed by MitoTracker staining in primary mouse chondrocytes transfected with siSirt4 or siCtrl. The inset in the images is shown as magnified images in the right row. Scale bars: 5 μm. (F) The MINA plug-in in ImageJ was used to analyze the mean branches number per network of mitochondria in primary mouse chondrocytes. n = 5, *p < 0.05. (G) The MINA plug-in in ImageJ was used to analyze the mean branches length of mitochondria in primary mouse chondrocytes. n = 5, ***p < 0.001. (H) NAO was used to determine the total mitochondria mass in chondrocytes transfected with siSirt4 or siCtrl by flow cytometry. (I) Relative mean fluorescence intensity of NAO in chondrocytes transfected with siSirt4 or siCtrl. n = 3, ****p < 0.0001. (J) JC-1 was used to detect the mitochondrial membrane potential in primary mouse chondrocytes transfected with siSirt4 or siCtrl. Scale bars: 50 μm. (K) Relative mean fluorescence intensity of Red to Green in primary mouse chondrocytes transfected with siSirt4 or siCtrl. n = 5, ****p < 0.0001. (L) ATP content in primary mouse chondrocytes transfected with siSirt4 or siCtrl. n = 3, ****p < 0.0001.

It is reported that the equilibrium between intracellular ROS generation and scavenging is controlled by antioxidant systems, especially enzymatic antioxidant systems^39^. In an endeavor to elucidate the role of Sirt4 in ROS production, we conducted an initial examination of the mRNA expression of genes associated with antioxidant enzymes in chondrocytes following Sirt4 knockdown. Our investigation unveiled that the mRNA expression levels of the transcription factor Nfe2l2, a crucial regulator of endogenous antioxidant defense mechanisms, along with the antioxidant enzymes Gpx1, Cat, and Sod2, showed no significant differences following Sirt4 knockdown in chondrocytes compared to the negative control (Fig. 5A-D).

Emerging evidence suggests that compromised or dysfunction mitochondria serve as the primary source of ROS production^41^. Therefore, we conducted a more detailed investigation into the impact of Sirt4 knockdown on mitochondrial function. Initially, mitochondria were stained with MitoTracker Red to enable visualization of their morphology. While normal chondrocytes exhibited an intact mitochondrial network with a tubular structure, the mitochondria in Sirt4 knockdown chondrocytes were markedly fragmented (Fig. 5E). The average number of branches per network decreased, and the mean branch length was shorter in Sirt4 knockdown chondrocytes compared to their normal counterparts, indicative of disrupted mitochondrial networking and excessive fragmentation of the mitochondrial network (Fig. 5F, G). Consistently, there was an increase in mitochondrial volume in siSirt4-treated chondrocytes when contrasted with the negative control, as detected by Nonyl acridine orange (NAO), a fluorescent probe used to assess mitochondrial mass (Fig. 5H, I). Subsequently, JC-1 was employed as a probe to evaluate mitochondrial membrane potential. The outcomes demonstrated a significant reduction in mitochondrial membrane potential in siSirt4-treated chondrocytes compared to the negative control (Fig. 5J, K). Adenosine triphosphate (ATP), the byproduct of mitochondrial oxidative respiration, serves as an indicator of mitochondrial health. Notably, the synthesis of ATP was compromised subsequent to Sirt4 knockdown in chondrocytes (Fig. 5L). In sum, these results underscore that Sirt4 knockdown induces mitochondrial dysfunction in chondrocytes, ultimately contributing to an escalation in ROS production.

### Sirt4 overexpression attenuates TBHP-induced mitochondrial dysfunction in chondrocytes

**Figure 6:**
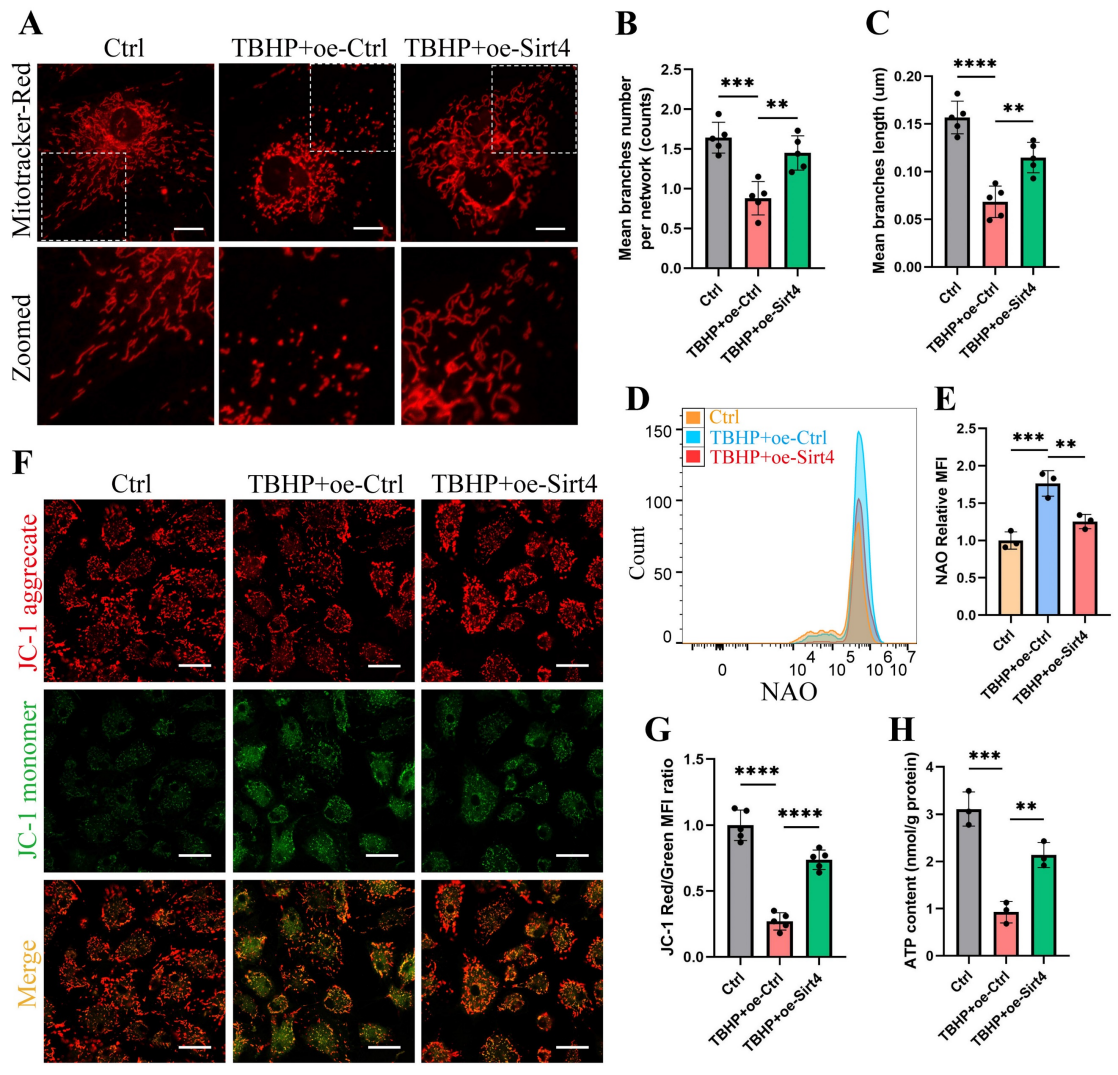
**Sirt4 overexpression attenuates TBHP-induced mitochondrial dysfunction in chondrocytes.** (A) Mitochondrial network was analyzed by MitoTracker staining in primary mouse chondrocytes transfected with oe-Sirt4 or oe-Ctrl plasmid and treated or untreated with TBHP (50 μM). The inset in the images is shown as magnified images in the bottom row. Scale bars: 5 μm. (B) The MINA plug-in in Image J was used for statistical analysis of mitochondrial morphology in Figure (A), and the mean branches number per network of mitochondria in primary mouse chondrocytes was obtained. n = 5, **p < 0.01, ***p < 0.001. (C) The MINA plug-in in Image J was used for statistical analysis of mitochondrial morphology in Figure (A), and the mean mitochondrial branch length in primary mouse chondrocytes was obtained through analysis. n = 5, **p < 0.01, ****p < 0.0001. (D) NAO was used to determine the total mitochondria quantity in chondrocytes transfected with oe-Sirt4 or oe-Ctrl and treated or untreated with TBHP by flow cytometry. (E) Relative mean fluorescence intensity of NAO in chondrocytes transfected with oe-Sirt4 or oe-Ctrl plasmid and treated or untreated with TBHP. n = 3, **p < 0.01, ***p < 0.001. (F) JC-1 was used to detect the mitochondrial membrane potential of primary mouse chondrocytes transfected with oe-Sirt4 or oe-Ctrl plasmid and treated or untreated with TBHP. Scale bars: 25 μm. (G) Relative mean fluorescence intensity of Red to Green in chondrocytes. n = 5, ****p < 0.0001. (H) ATP content of primary mouse chondrocytes transfected with oe-Sirt4 or oe-Ctrl plasmid and treated or untreated with TBHP. n = 3, **p < 0.01, ***p < 0.001.

We proceeded to substantiate the aforementioned findings within TBHP-induced senescent chondrocytes. Initially, we employed MitoTracker Red to label mitochondria and conducted a thorough examination of mitochondrial network morphology through confocal microscopy. Our observations revealed discernible damage to the mitochondrial network in senescent chondrocytes induced by TBHP, characterized by an increase in mitochondrial fragments. Remarkably, upon Sirt4 overexpression, a substantial reduction in mitochondrial fragmentation was achieved, consequently facilitating a partial restoration of the mitochondrial network's integrity (Fig. 6A-C). In addition, NAO assay showed that Sirt4 overexpression abolished TBHP-mediated increase in total mitochondrial volume in chondrocytes (Fig. 6D, E). Next, JC-1 assay showed that the mitochondrial membrane potential was significantly decreased in senescent chondrocytes, and Sirt4 overexpression could restore the polarization state of chondrocytes (Fig. 6F, G). In parallel, we detected the content of ATP in chondrocytes. The results showed that the content of ATP in senescent chondrocytes was significantly reduced compared with the negative control group, and the intracellular ATP was significantly increased after Sirt4 overexpression (Fig. 6H). Collectively, these results suggested that the number of dysfunction or damaged mitochondria in senescent chondrocytes increased, leading to the increase of ROS production. Notably, Sirt4 overexpression restored mitochondrial homeostasis and reduced ROS level (Supplementary Fig. 4) in senescent chondrocytes.

### Sirt4 overexpression attenuates ROS-mediated senescence of chondrocytes via mitophagy

**Figure 7:**
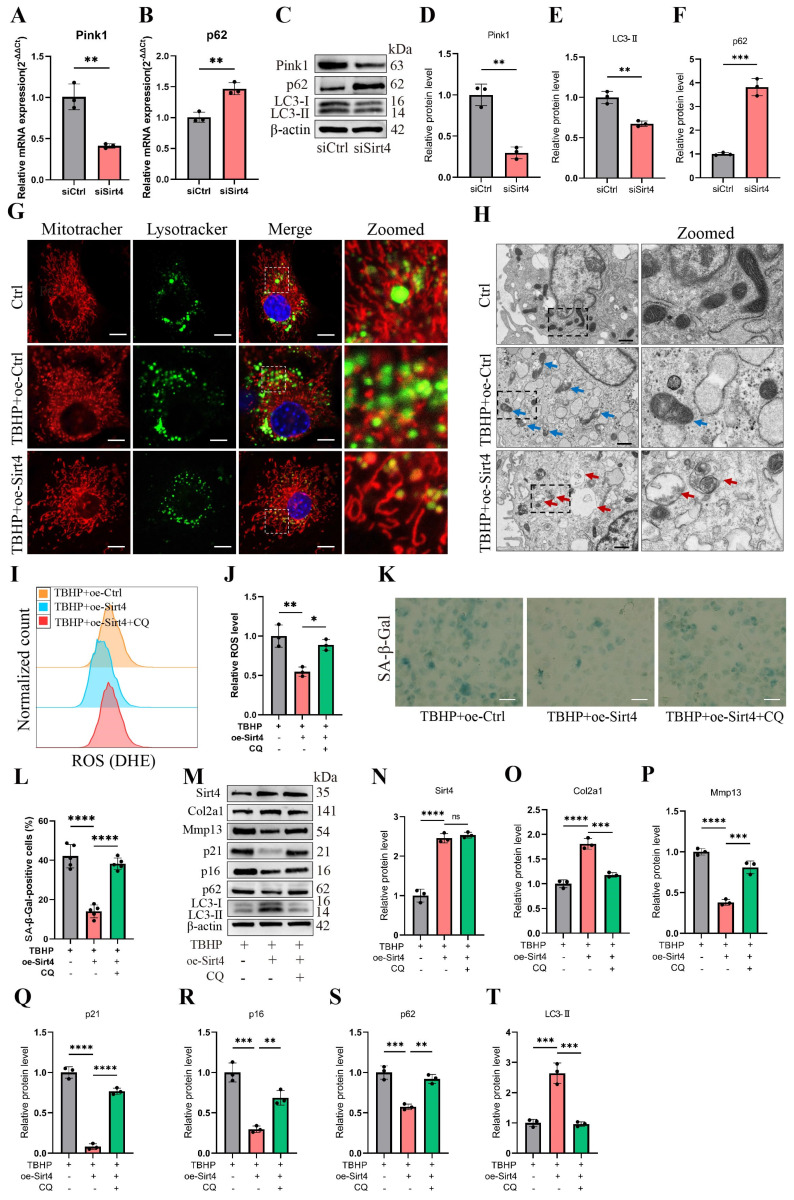
** Sirt4 overexpression attenuates ROS-mediated senescence of chondrocytes and cartilage degradation via enhancing mitophagy.** (A, B) Relative mRNA expression level of Pink1 and p62 in primary mouse chondrocytes transfected with siSirt4 compared to siCtrl. β-actin was used as normalization control. n = 3, **p < 0.01. (C-F) Western blotting analysis of the protein expression level of Pink1, LC3 and p62 in primary mouse chondrocytes transfected with siSirt4 compared to siCtrl. Band intensity relative to β-actin. n = 3, **p < 0.01, ***p < 0.001. (G) Primary mouse chondrocytes were transfected with oe-Ctrl plasmid or oe-Sirt4 plasmid. The cells were subsequently cultured for 2 days with or without TBHP (50 μM). The chondrocytes were stained with MitoTracker Red (100 nM, 30 min) and LysoTracker Green (75 nM, 30 min) to label the mitochondria and lysosomes, respectively. The co-localization of mitochondria and lysosomes was detected by laser scanning confocal microscopy. The inset box in the images is shown as magnified images in the rightmost row. Scale bar = 5 μm. (H) Primary mouse chondrocytes were transfected with oe-Ctrl plasmid or oe-Sirt4 plasmid. The cells were subsequently cultured for 2 days with or without TBHP (50 μM). The mitochondrial morphology of each group was analyzed by transmission electron microscope (TEM). The blue arrow represents impaired mitochondria and the red arrow represents mitophagy or autophagolysosome. The inset box in the images is shown as magnified images in the right row. Scale bar: 10 μm. (I, J) The chondrocytes were pretreated with CQ, followed by treatment with TBHP and with or without transfection with oe-Sirt4 plasmid. Flow cytometer analysis of DHE fluorescence measuring levels of intracellular O2^•-^ in primary mouse chondrocytes of different treatment groups. The bar graph shows the fluorescence intensity of the DHE. n = 3, *p < 0.05, **p < 0.01. (K, L) The chondrocytes were pretreated with CQ, followed by treatment with TBHP and with or without transfection with oe-Sirt4 plasmid. SA-β-Gal staining and quantification of SA-β-Gal positivity in primary mouse chondrocytes of different treatment groups. n = 5, ****p < 0.0001. Scale bars: 50 μm. (M-T) The chondrocytes were pretreated with CQ, followed by treatment with TBHP and with or without transfection with oe-Sirt4 plasmid. Protein levels of Sirt4, Col2a1, Mmp13, p16, p21, p62 and LC3-II were analyzed in primary mouse chondrocytes of different treatment groups. Band intensity relative to β-actin. n = 3, **p < 0.05, ***p < 0.001, ****p < 0.0001.

We proceeded to delve into the mechanism through which Sirt4 is implicated in the regulation of mitochondrial dysfunction. MQC, a highly orchestrated regulatory system, plays a crucial role in harmonizing diverse mitochondrial biofunctions and upholding mitochondrial homeostasis. MQC encompasses three principal biological processes: mitochondrial biogenesis (mitogenesis), mitochondrial dynamics, and mitophagy^18^. To begin, we examined the expression levels of genes related to these processes subsequent to Sirt4 knockdown in chondrocytes. RT-qPCR analysis revealed an elevation in the mRNA expression levels of Drp1 and p62, coupled with a significant reduction in the expression of Pink1 in Sirt4 knockdown chondrocytes as compared to the negative control (Fig. 7A, B and Supplementary Fig. 5). Nevertheless, the expression level of Pgc1α, Bnip3, and Opa1 displayed no notable distinctions (Supplementary Fig. 5). These findings imply an increase in the processes of mitochondrial fission while a decrease in mitophagy within Sirt4 knockdown chondrocytes. Furthermore, following Sirt4 knockdown, the protein level of p62 exhibited an augmentation, whereas the protein levels of LC3-II and Pink1 displayed a reduction, signifying an impediment in mitophagy flux (Fig. 7C-F).

Considering the significance of mitophagy for maintaining mitochondrial equilibrium and ROS levels in chondrocytes^42^,^43^, we posited that Sirt4 modulates mitochondrial homeostasis in chondrocytes via mitophagy, thus governing ROS generation and cellular senescence.

To scrutinize this hypothesis, we initially assessed the expression of autophagy-related genes in TBHP-treated chondrocytes. The outcomes unveiled an increase in p62 levels and a reduction in LC3-II levels in a concentration-dependent manner, indicating a blockade in autophagy flux within TBHP-induced senescent chondrocytes (Supplementary Fig. 6). Subsequently, MitoTracker and LysoTracker were employed for fluorescence colocalization analysis of mitochondria and lysosomes, respectively. The results indicated a significant increase in the colocalization of mitochondria and lysosomes in TBHP-induced senescent chondrocytes following Sirt4 overexpression (Fig. 7G). To further investigate, we employed transmission electron microscopy to observe the morphology and structure of mitochondria in chondrocytes. Our observations revealed an augmented presence of swollen and damaged mitochondria in TBHP-induced senescent chondrocytes when contrasted with negative control cells. However, upon Sirt4 overexpression, a notable reduction in damaged mitochondria was observed in comparison to senescent chondrocytes. Additionally, the presence of mitochondrial autophagosomes was detected (Fig. 7H), suggesting that Sirt4 overexpression might enhance the levels of mitophagy. These findings collectively suggest that Sirt4 overexpression could stimulate mitophagy in senescent chondrocytes.

To further substantiate whether Sirt4 can dampen ROS production and chondrocyte senescence by promoting mitophagy, we employed Chloroquine (CQ), an autophagy inhibitor that impedes the fusion of autophagosomes with lysosomes, to obstruct autophagy and assess its effect on ROS production and cellular senescence. Initially, Sirt4 overexpression yielded a reduction in ROS levels within TBHP-induced senescent chondrocytes when compared with senescent chondrocytes. However, this ameliorative effect was negated when mitophagy was suppressed by CQ (Fig. 7I, J). Subsequently, the SA-β-gal assay demonstrated a notable decrease in chondrocyte senescence in the group subjected to TBHP and oe-Sirt4 plasmid treatment, as compared to the TBHP-only group. Nonetheless, the introduction of CQ to inhibit mitophagy led to a marked increase in the positive rate of SA-β-gal staining, indicating the attenuation of Sirt4 overexpression's inhibitory effect on chondrocyte senescence (Fig. 7K, L). Subsequent Western blotting analyses were conducted to assess the expression of typical ECM markers, autophagy markers, and senescence markers. The results unveiled that when mitophagy was impeded, the protein expression levels of Mmp13, p16, p21, and p62 escalated, while the expression levels of Col2a1 and LC3-II diminished (Fig. 7M-T). In combination, these findings suggest that Sirt4 overexpression counteracts TBHP-induced chondrocyte senescence and extracellular matrix (ECM) degradation by fostering mitophagy.

### Pink1 is a downstream regulator in Sirt4-mediated mitophagy in chondrocytes

**Figure 8:**
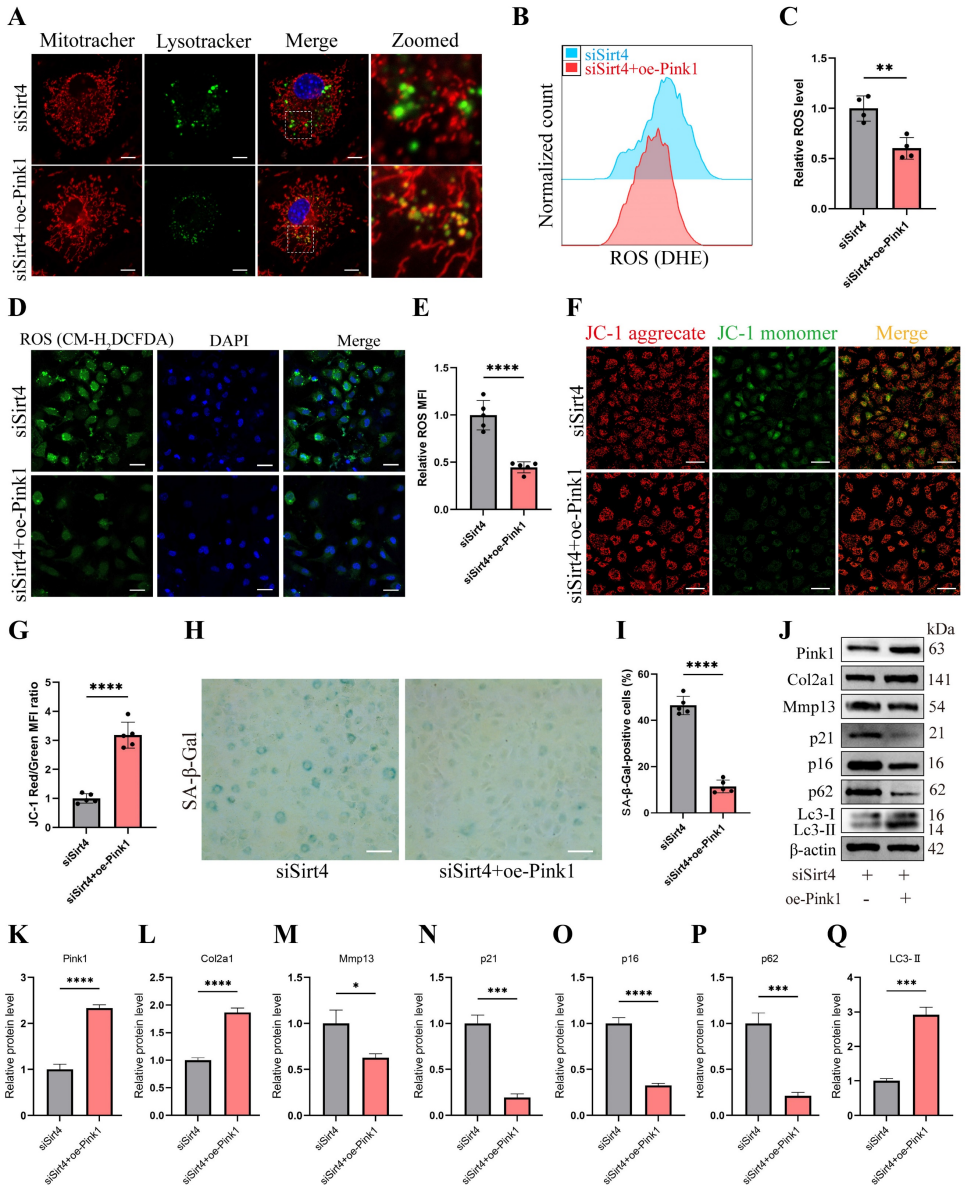
** Sirt4 mediates mitophagy via Pink1 in chondrocytes.** (A) Double-labeled fluorescence staining by LysoTracker Green (75 nM, 30 min) and MitoTracker Deep Red (100 nM, 30 min) dye in chondrocytes transfected with siSirt4 and with or without oe-Pink1 plasmid. The inset box in the images is shown as magnified images in the right row. Scale bar: 5 μm. (B, C) Flow cytometer analysis of DHE fluorescence measuring levels of intracellular O_2_^•-^ in primary mouse chondrocytes transfected with siSirt4 and with or without oe-Pink1 plasmid. The bar graph shows the fluorescence intensity of the DHE. n = 4, **p < 0.01. (D, E) Confocal imaging analysis of CM-H_2_DCFDA fluorescence measuring levels of intracellular H_2_O_2_ in primary mouse chondrocytes transfected with siSirt4 and with or without oe-Pink1 plasmid. The bar graph shows the fluorescence intensity of the CM-H_2_DCFDA. n = 5, ****p < 0.0001, Scale bars: 50 μm. (F) JC-1 was used to detect the mitochondrial membrane potential of primary mouse chondrocytes transfected with siSirt4 and with or without oe-Pink1 plasmid. Scale bars: 50 μm. (G) Relative mean fluorescence intensity of Red to Green. n = 5, ****p < 0.0001. (H, I) SA-β-Gal staining and quantification of SA-β-Gal positivity in primary mouse chondrocytes transfected with siSirt4 and with or without oe-Pink1 plasmid. n = 5, ****p < 0.0001. Scale bars: 50 μm. (J-Q) Protein levels of Pink1, Col2a1, Mmp13, P16, P21, P62 and Lc3-II were analyzed in primary mouse chondrocytes transfected with siSirt4 and with or without oe-Pink1 plasmid using western blotting. Band intensity relative to β-actin. n = 3, *p < 0.05, ***p < 0.001, ****p < 0.0001.

RT-qPCR and Western blotting analyses revealed a significant reduction in the expression of Pink1, a pivotal protein orchestrating mitophagy, in Sirt4 knockdown chondrocytes (Fig. 7A, C). To further substantiate whether Sirt4 triggers mitophagy through the upregulation of Pink1 expression, chondrocytes were exposed to a plasmid encoding Pink1 (oe-Pink1) subsequent to Sirt4 knockdown. The efficiency of the oe-Pink1 plasmid transduction was confirmed in primary mouse chondrocytes through RT-qPCR and Western blot analyses (Supplementary Fig. 7). Initially, we performed double-labeled fluorescence staining employing LysoTracker Green and MitoTracker Deep Red in chondrocytes transfected with siSirt4, with or without the oe-Pink1 plasmid. The outcomes demonstrated a noteworthy augmentation in mitochondrial and lysosomal colocalization within chondrocytes concurrently treated with siSirt4 and the oe-Pink1 plasmid when compared to those treated with siSirt4 alone. This suggested that the overexpression of Pink1 counteracted the hampered mitophagy in Sirt4 knockdown chondrocytes (Fig. 8A).

Subsequently, intracellular ROS levels were assessed, and it was observed that chondrocytes treated with both siSirt4 and the oe-Pink1 plasmid exhibited a marked reduction in intracellular ROS levels in comparison to those treated solely with siSirt4 (Fig. 8B-E). Furthermore, the measurement of mitochondrial membrane potential revealed that Pink1 overexpression reinstated the diminished mitochondrial membrane potential induced by Sirt4 knockdown in chondrocytes (Fig. 8F, G). Additionally, SA-β-gal staining indicated that the concurrent treatment of chondrocytes with siSirt4 and the oe-Pink1 plasmid significantly decreased the positive rate of senescent chondrocytes when contrasted with those treated solely with siSirt4 (Fig. 8H, I). Consequently, we proceeded to scrutinize the protein expressions of typical ECM markers, autophagy markers, and senescence markers in chondrocytes. Enhanced protein expression levels of Pink1, Col2a1, and LC3-II, alongside diminished Mmp13, p62, p16, and p21 levels, were discerned in chondrocytes concurrently treated with siSirt4 and the oe-Pink1 plasmid in comparison to those treated solely with siSirt4 (Fig. 8J-Q). Collectively, these compelling findings illuminate that Sirt4 knockdown curtails mitophagy through the downregulation of Pink1 expression. The mitophagy inhibition further stimulates ROS production, ECM degradation, and contributes to chondrocyte senescence.

### Gene therapy with Sirt4 alleviates the progression of OA in mouse OA model

**Figure 9:**
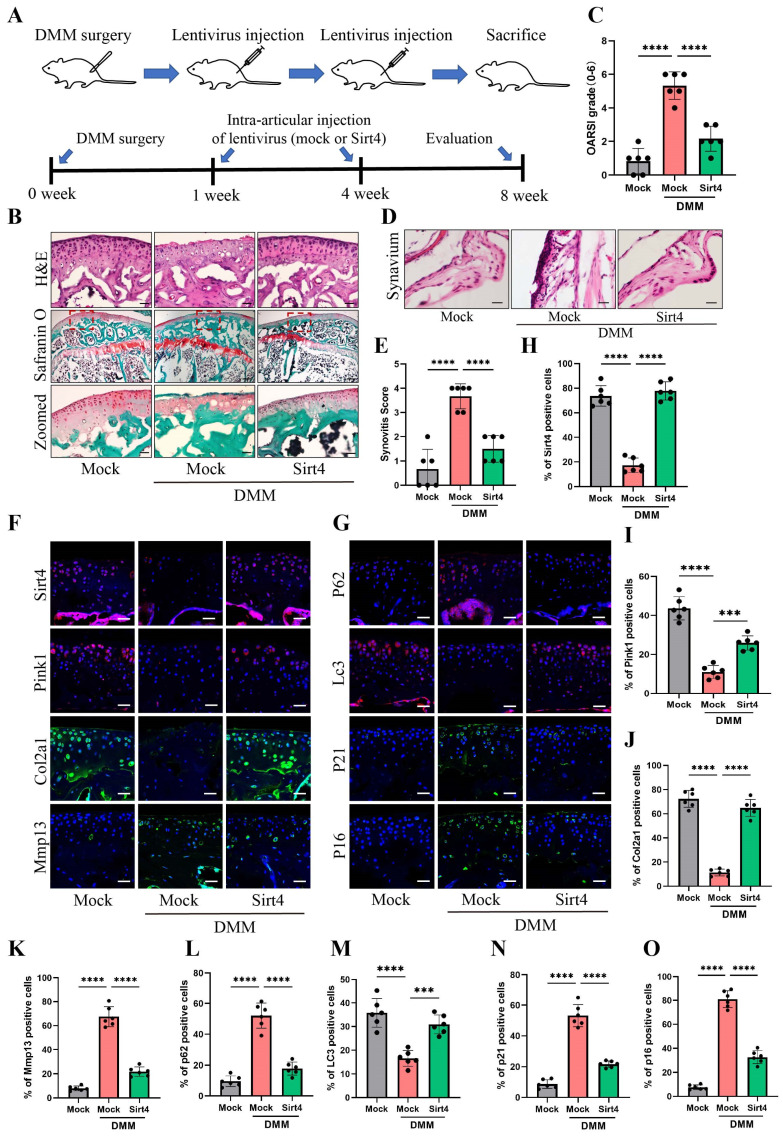
** Gene therapy with a lentiviral vector encoding Sirt4 in a mouse model for OA.** (A) Schematic of the time course used for the DMM-induced in vivo OA experiments. (B) H&E staining was performed to observe the cell morphology and tissue integrity in the articular cartilage tissues of the mouse knee undergoing sham or DMM surgery. Likewise, the middle and bottom figures show Safranin O and fast green staining of articular cartilage tissues from mice that underwent sham or DMM surgery is indicated. Safranin O and fast green staining demonstrated OA progression through the 8-week time course in the medial tibial plateau. Scale bar: 50μm or 200μm (middle figure). (C) OARSI scores of the medial tibial plateau of sham or DMM mice. Data are expressed as means, and the error bars indicate the standard deviation. n = 6, ****p < 0.0001. (D, E) The representative images and scores of synovitis of mice that underwent sham or DMM surgery. n = 6, ***p < 0.001, ****p < 0.0001. (F-G) Representative images for Sirt4, Pink1, Col2a1, Mmp13, p21, p16, p62 and Lc3 immunofluorescent staining in cartilage tissues obtained from sham or DMM mouse knees. Scale bar: 50 μm. (H-O) The bar graphs show quantification (%) of the Sirt4, Pink1, Col2a1, Mmp13, p21, p16, p62 and Lc3-positive cells from total cell population per field in immunofluorescent staining sections. n = 6, **p < 0.01, ***p < 0.001, ****p < 0.0001.

To explore the viability of Sirt4 as a therapeutic target for OA, we employed a lentiviral vector encoding mouse Sirt4 in conjunction with the surgical destabilization of the medial meniscus (DMM) model for in vivo animal studies. Our investigation aimed to assess the impact of Sirt4 overexpression on the homeostasis of cartilage matrix and the senescence of chondrocytes within the DMM-induced OA mouse model. The lentivirus encoding Sirt4 was administered into the knee articular cavity one week and four weeks after DMM surgery (Fig. 9A). Cartilage degradation was evaluated eight weeks post DMM surgery within the weight-bearing region of the medial tibial plateau, employing HE staining, safranin O staining, and the OARSI grade^35^. Notably, at eight weeks post DMM surgery, HE staining revealed a loss of cartilage matrix in the superficial region of the mouse knee cartilage within the DMM group. However, injection of Sirt4-lentivirus successfully mitigated the DMM-induced cartilage matrix destruction (Fig. 9B). Furthermore, the OARSI score in the DMM mice injected with Sirt4-lentivirus exhibited a significant reduction in comparison to the mock vector-injected DMM group (Fig. 9B, C). Observations at the eight-week post-DMM interval further revealed a thicker lining layer and heightened proliferation of synovial tissue within the DMM group, accompanied by a heightened presence of inflammatory cells. Intriguingly, the introduction of the lentivirus encoding Sirt4 significantly abated the synovitis triggered by the DMM surgery (Fig. 9D). Corroborating the histological observations, the Sirt4-lentivirus injected group exhibited notably lower synovitis scores eight weeks post DMM surgery in comparison to the DMM group (Fig. 9E and Supplementary Table 3). Subsequently, we assessed whether intra-articular injection of the Sirt4 lentivirus affected the expression levels of Pink1, key ECM markers, autophagy-related genes, and senescence markers within cartilage tissues subsequent to DMM surgery. While Sirt4 expression was almost undetectable in the DMM-induced OA knee, it was notably augmented in mice injected with the lentivirus encoding mouse Sirt4. Impressively, the administration of the Sirt4-encoding lentivirus not only suppressed the DMM-induced upregulation of Mmp13, p62, p21, and p16, but also averted the DMM-induced downregulation of Pink1, Col2a1, and LC3 (Fig. 9F-O). Collectively, these findings suggest that intra-articular delivery of the Sirt4 gene could offer a promising avenue for OA treatment. This approach appears to effectively preserve cartilage integrity, stimulate autophagy, and curtail chondrocyte senescence within the OA microenvironment in vivo.

## Discussion

**Figure 10:**
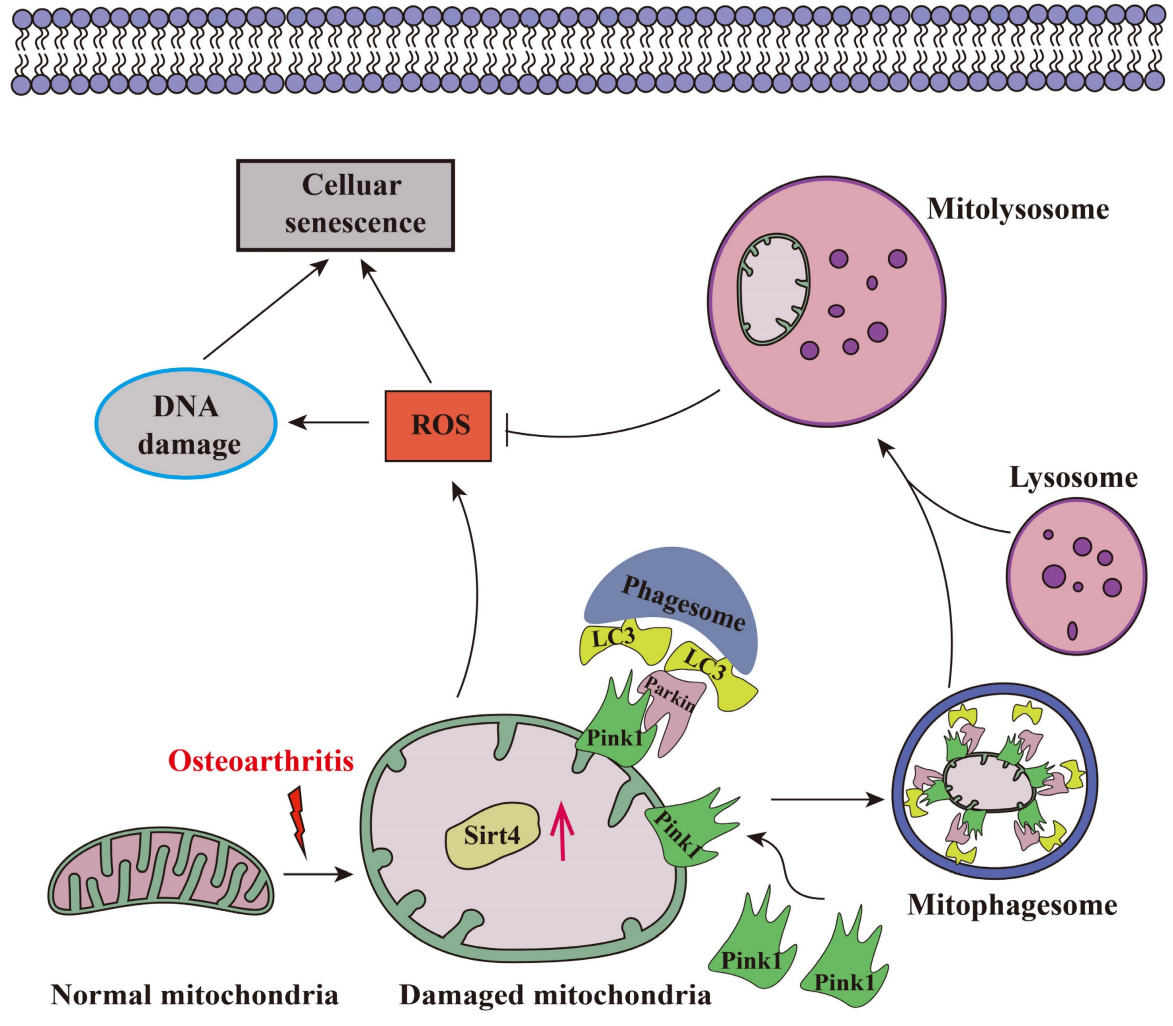
Schematic representation of mechanisms by which Sirt4 mediates chondrocyte senescence and OA development.

Aging stands as an important factor in the progression of OA, characterized by cellular senescence and the gradual decline in tissue and organ functionality over time^44^. Recent studies have unveiled chondrocyte senescence as a prevalent molecular mechanism underlying both age-associated and post-traumatic OA manifestations, yet the intricate molecular pathways governing this phenomenon remain veiled in uncertainty^45^. In our current investigation, we shed light on the contributory role of Sirt4 downregulation in promoting chondrocyte senescence and subsequent cartilage degradation. Mechanistically, we elucidate that diminished Sirt4 levels hinder Pink1-mediated mitophagy, leading to the accumulation of dysfunctional mitochondria and the generation of ROS. These ROS, in turn, play a pivotal role in triggering cellular senescence, as illustrated in Fig. 10. In addition, Sirt4 effectively reduced the progression of DMM-induced OA in vivo. Our findings highlight the latent therapeutic potential of Sirt4, positioning it as a promising target for mitigating OA pathogenesis. This discovery opens new avenues for therapeutic interventions in the realm of OA research.

Recent advancements underscore Sirtuins as promising therapeutic targets for aging-related ailments, including type 2 diabetes, OA, and neurodegenerative disorders^46^,^47^. Notably, Matsuzaki et al. revealed that Sirt1 loss in chondrocytes accelerates OA progression in aging mice^48^. Conversely, SIRT1 activators, such as SRT2104, have exhibited longevity-promoting effects, preserving bone and muscle mass in mouse models^49^. Fu et al. illuminated Sirt3's involvement in age-related cartilage redox regulation alterations and its protective role against early-stage OA^50^. Furthermore, Nagai et al. established the significance of SIRT6 in preventing cellular senescence, DNA damage, and telomere dysfunction in human chondrocytes, underscoring its protective function^51^. Korogi et al. provided compelling evidence by demonstrating that Sirt7 knockout mice display resistance to aging-associated OA development^52^. Moreover, Parik et al. highlighted mitochondrial dSirt4 as a key factor determining longevity, emphasizing that its loss accelerates aging processes^53^. In our present study, we contribute to this growing body of knowledge by revealing that Sirt4 knockdown induces chondrocyte senescence. Intriguingly, our findings also indicate that Sirt4 overexpression shields chondrocytes from senescence induced by TBHP, further emphasizing the therapeutic potential of modulating Sirt4 in OA research.

Elevated levels of ROS play a pivotal role in driving chondrocyte senescence through oxidative stress. The role of Sirt4 in modulating ROS remains a topic of debate^54^. In a study investigating angiotensin II-induced cardiac hypertrophy in mice, both Sirt4 overexpression and knockout led to increased and decreased ROS levels, respectively, in both the cardiomyocytes and mitochondria^55^. On the contrary, SIRT4 overexpression demonstrated a protective effect by enhancing mitochondrial membrane potential and curbing ROS production in podocytes, suppressing apoptosis^56^. Furthermore, Sirt4 exhibited a safeguarding role in vascular endothelial cells, shielding them from injury induced by oxidized low-density lipoprotein^57^. Ding et al.'s work in bovine mammary epithelial cells showed that SIRT4 depletion resulted in heightened ROS generation^58^. Our study aligns with these findings, demonstrating that Sirt4 knockdown in chondrocytes increased ROS production, while Sirt4 overexpression countered TBHP-induced ROS levels. Nevertheless, despite overexpressing Sirt4, a complete rescue of the cells was not achieved. Notably, the expression levels of Sirt3 and Sirt5 displayed a substantial decrease in senescent chondrocytes induced by TBHP. This observation may provide an explanation for the incomplete rescue observed in senescent chondrocytes following Sirt4 overexpression.

Mitochondria, recognized as the primary ROS source, play a pivotal role in chondrocyte function. Dysfunctional mitochondria elevate ROS production, decrease ATP synthesis, and induce matrix metalloproteinase (MMP) synthesis, culminating in cellular senescence and OA onset^59^-^61^. Targeting mitochondria for anti-aging interventions emerges as a promising therapeutic avenue for OA prevention in the elderly^9^. Mitochondrial dynamics, governed by fusion and fission processes, crucially maintain mitochondrial quality^62^. Fusion proteins MFN1/2 and OPA1 enhance mitochondrial content exchange and restore function, while fission proteins DRP1 and FIS1 fragment mitochondria^18^. Notably, DRP1 upregulation, correlating with mitochondrial damage, is observed in human OA cartilage^63^. SIRT4, known to interact with OPA1, promotes fusion, sustaining mitochondrial integrity^64^. Conversely, SIRT4 depletion induces fission by enhancing ERK-regulated Drp1 phosphorylation^65^. Our study aligns with these findings, indicating that Sirt4 knockdown upregulates Drp1, potentially leading to mitochondrial network fragmentation and dysfunction. These insights underscore the critical role of Sirt4 in mitochondrial dynamics, shedding light on potential avenues for therapeutic interventions in chondrocyte senescence and OA.

Mitophagy, another crucial mitochondrial quality control mechanism, plays a pivotal role in eliminating damaged mitochondria and curbing ROS production^66^. Aged chondrocytes exhibit reduced mitophagy, leading to dysfunctional mitochondria accumulation, heightened ROS generation, and a senescent phenotype^67^,^68^. Despite this, the intricate regulatory mechanisms and mitophagy modulators remain incompletely understood^42^. The PTEN-induced kinase 1 (PINK1)-Parkin pathway has emerged as a central signaling pathway in mitophagy. Recent studies have highlighted its vital role in limiting ROS production and enhancing cell survival in OA chondrocytes^42^. For example, Ansari et al. underscored Pink1-mediated mitophagy as a critical mechanism for limiting ROS production and bolstering OA chondrocyte survival^23^. Kang et al. demonstrated PARKIN's role in averting ROS-induced apoptosis by inducing mitophagy and enhancing antioxidant defenses in intervertebral endplate chondrocytes^68^. However, divergent effects have been noted, with Shin et al. suggesting that PINK1/PARKIN-mediated mitophagy can lead to the death of OA chondrocytes^69^. The intricate interplay of mitophagy's effects on chondrocyte survival and function may be influenced by the level of mitophagy at specific stages and the OA stage. Lang et al. have indicated that SIRT4 plays a regulatory role in mitochondrial quality control and mitophagy^64^. Li et al. pointed to Sirt4's ability to activate autophagy through the upregulation of the p53 signaling pathway^70^. In our study, we elucidated the suppressed mitophagy in senescent chondrocytes and revealed that Sirt4 overexpression can restore autophagic flux and mitophagy by enhancing Pink1 expression, ultimately reducing mitochondrial ROS production in senescent chondrocytes. Inhibition of mitophagy with CQ diminishes Sirt4's protective effect on mitochondrial function and chondrocyte senescence, underscoring mitophagy's pivotal role in mediating Sirt4's beneficial effects.

While our study has demonstrated that Sirt4 mitigates mitochondrial dysfunction and senescence in TBHP-treated chondrocytes through the Pink1-mediated mitophagy signaling pathway, there remains a limitation in our research. Specifically, the mechanism by which Sirt4 regulates the level of Pink1 is still unclear. Sirt4, an enzyme localized in mitochondria, exhibits a versatile repertoire of activities, including deacetylase, ADP-ribosyltransferase, and lipoylase functions^71^. Notably, the posttranslational modification of acetylation plays a pivotal role in the intricate regulation of autophagy^72^,^73^. Huang et al. suggested that the acetylation of SCFD1 impedes autophagic flux, while SIRT4-mediated deacetylation of SCFD1 restores this process^71^. Consequently, SIRT4 may also function as a deacetylase in our model. Additionally, Li et al. indicated that SIRT3 regulates mitophagy in liver fibrosis through deacetylation of PINK1/NIPSNAP1^74^. In our research, Sirt4 may regulate Pink1 by deacetylating it, thus contributing to the regulation of mitophagy and mitochondrial dysfunction. However, the deacetylation activity of Sirt4 and its regulatory mechanism on Pink1 in chondrocytes necessitate further investigation.

This study significantly augments our understanding of Sirt4's role in cellular senescence and OA advancement. Our data unequivocally indicates the downregulation of Sirt4 and Pink1 during chondrocyte aging and OA progression. Furthermore, we have demonstrated that Sirt4 plays a crucial role in inhibiting mitochondrial dysfunction by facilitating Pink1-mediated mitophagy in OA, thereby mitigating chondrocyte senescence and cartilage degradation. Consequently, the upregulation of Sirt4 expression to enhance Pink1 levels within OA cartilage emerges as a promising therapeutic strategy for OA and age-related disorders arising from compromised mitophagy and mitochondrial dysfunction.

## Supplementary Material

Supplementary figures and tables.

## Abbreviations

OA: Osteoarthritis
Sirt4: Sirtuin 4
ROS: Reactive oxygen species
SASP: Senescence-associated secretory phenotype
ATP: Adenosine triphosphate
ECM: Extracellular matrix
MQC: Mitochondrial quality control
siRNA: Small interference RNA
CCK‑8: Cell Counting Kit‑8
SA-β-Gal: Senescence-associated β-galactosidase
NAO: 10-N-nonyl-acridine orange
ΔΨm: Mitochondrial membrane potential
TEM: Transmission electron microscopy
DMM: Destabilization of the medial meniscus
HE: Hematoxylin and eosin
OARSI: Osteoarthritis Research Society International
TBHP: Tert-butyl hydroperoxide
MMP: Matrix metalloproteinase
