# Supplementary material for: Sirtuin 4 (Sirt4) downregulation contributes to chondrocyte senescence and osteoarthritis via mediating mitochondrial dysfunction
Source: Int J Biol Sci. 2024 Jan 27;20(4):1256–78. doi: 10.7150/ijbs.85585 (PMC10878156; doi:10.7150/ijbs.85585)
Supplement: Supplementary file 1 — Supplementary figures and tables. [file ijbsv20p1256s1.pdf]

SUPPLEMENTARY MATERIAL

Sirtuin 4 (Sirt4) downregulation contributes to chondrocyte senescence and osteoarthritis via mediating mitochondrial dysfunction

Shiyuan Lin et al.

*Corresponding author. Email: hxjys888@hotmail.com (Xinjia Hu); johnniehuading@163.com (Huading Lu)

Supplementary materials

Supplementary Fig. 1. Model of TBHP-induced cellular senescence in chondrocytes.

Supplementary Fig. 2. The expression level of Sirt3 and Sirt5 in senescent chondrocytes.

Supplementary Fig. 3. The expression level of TNFα and Mmp10 after Sirt4 knockdown in chondrocytes.

Supplementary Fig. 4. Sirt4 overexpression decreases TBHP-induced ROS production in chondrocytes.

Supplementary Fig. 5. The mRNA expression level of genes relative to mitochondrial quality control after Sirt4 knockdown in chondrocytes.

Supplementary Fig. 6. Autophagy is inhibited in TBHP-induced cellular senescence model in chondrocytes.

Supplementary Fig. 7. Transfection efficiency of plasmid encoding Pink1 in primary mouse chondrocytes.

Supplementary Table 1. List of primers used in the present study.

Supplementary Table 2. siRNAs used in the current study.

Supplementary Table 3. Synovitis score.

Supplementary figures and figure legends

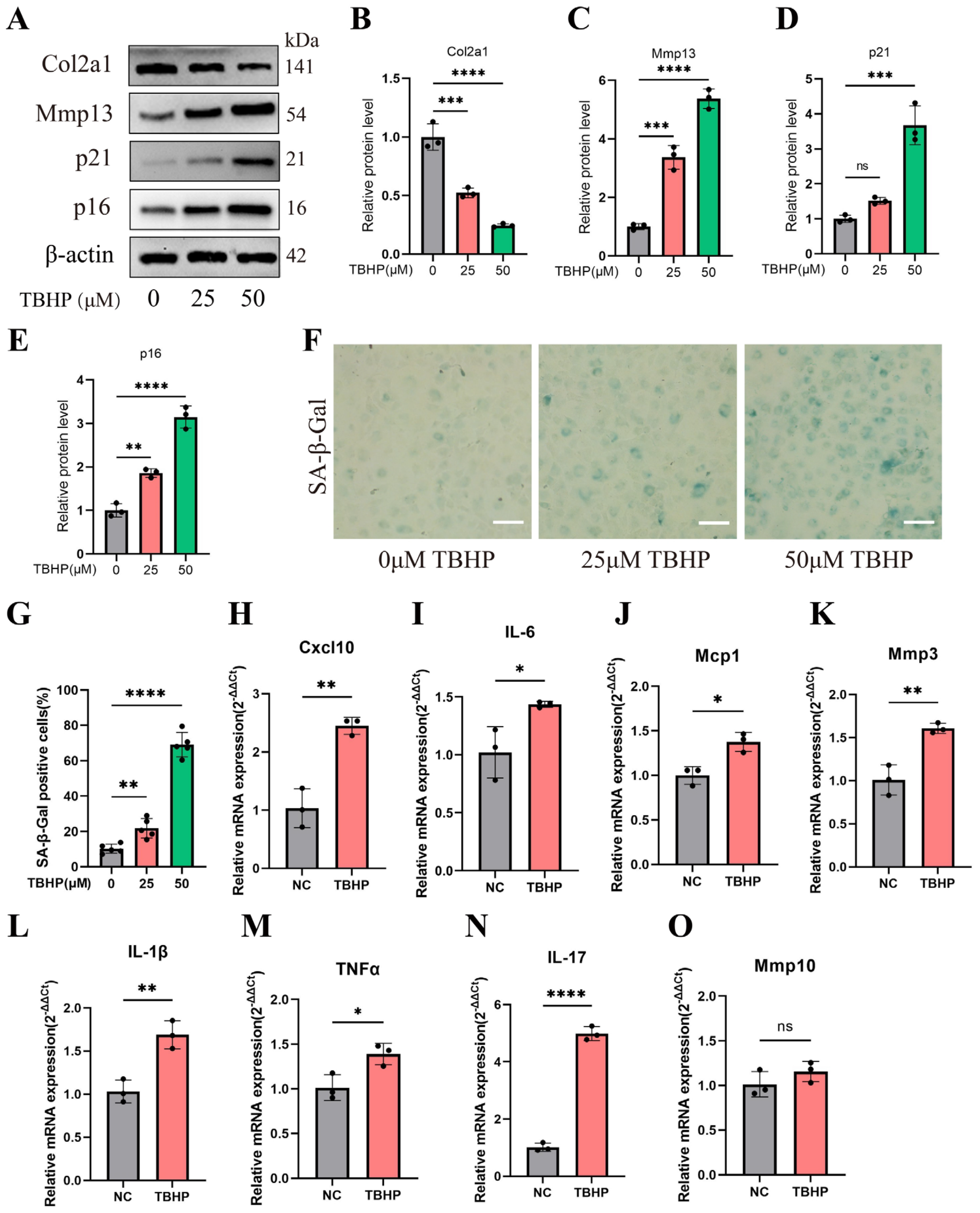

Supplementary Figure 1. Model of TBHP-induced cellular senescence in chondrocytes. (A-E) Western blotting analysis of Col2a1, Mmp13, p21 and p16 protein levels in primary mouse chondrocytes treated with various concentrations of

TBHP for 48h. β-actin was used as normalization control. n=3, **p < 0.01, ***p < 0.001, ****p < 0.0001. (F, G) SA-β-Gal staining of primary mouse chondrocytes treated with a concentration gradient of TBHP for 48 h. The SA-β-gal positive cells were quantified using ImageJ. Scale bar = 100 μm. n = 5, *p < 0.05, ****p < 0.0001. (H-O) qRT-PCR analysis of Cxcl10, IL-6, Mcp1, Mmp3, IL-1β, TNFα, IL-17 and Mmp10 mRNA expression levels in primary mouse chondrocytes treated with or without 50 μM TBHP for 48 h. β-actin was used as normalization control. n = 3, *p < 0.05, **p < 0.01. ****p < 0.0001.

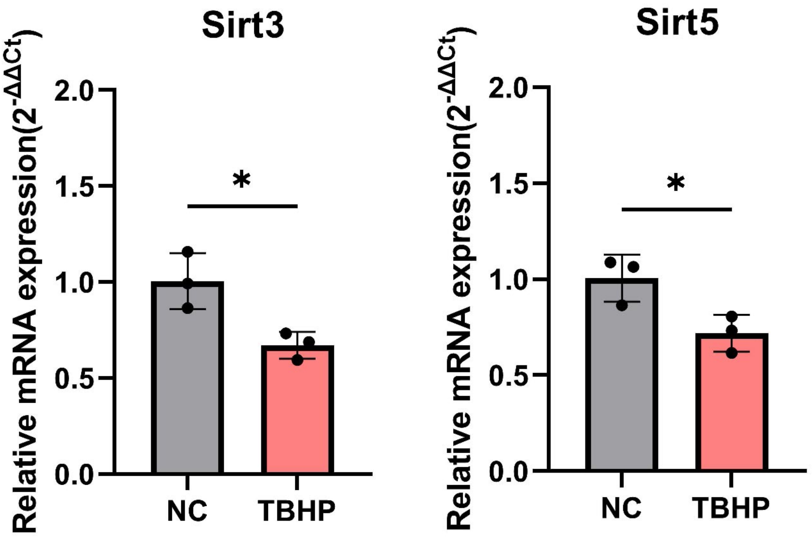

Supplementary figure 2. The expression level of Sirt3 and Sirt5 in senescent chondrocytes. (A, B) RT-qPCR analysis of the expression level of Sirt3 and Sirt5 in primary mouse chondrocytes treated with THHP. β-actin was used as normalization control. n = 3. *p < 0.05

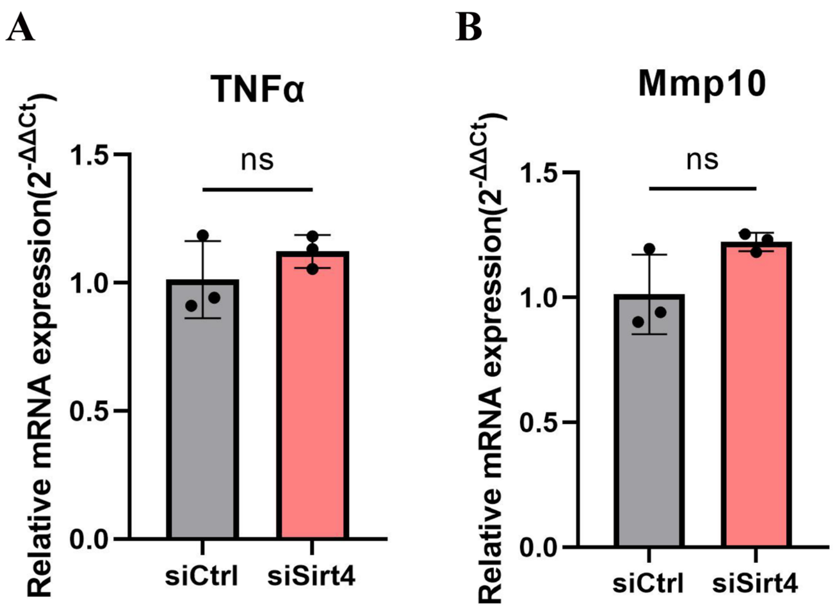

Supplementary figure 3. The expression level of TNFα and Mmp10 after Sirt4 knockdown in chondrocytes. (A, B) RT-qPCR analysis of the expression level of TNFα and Mmp10 in primary mouse chondrocytes transfected with siSirt4 or siCtrl. β-actin was used as normalization control. n = 3.

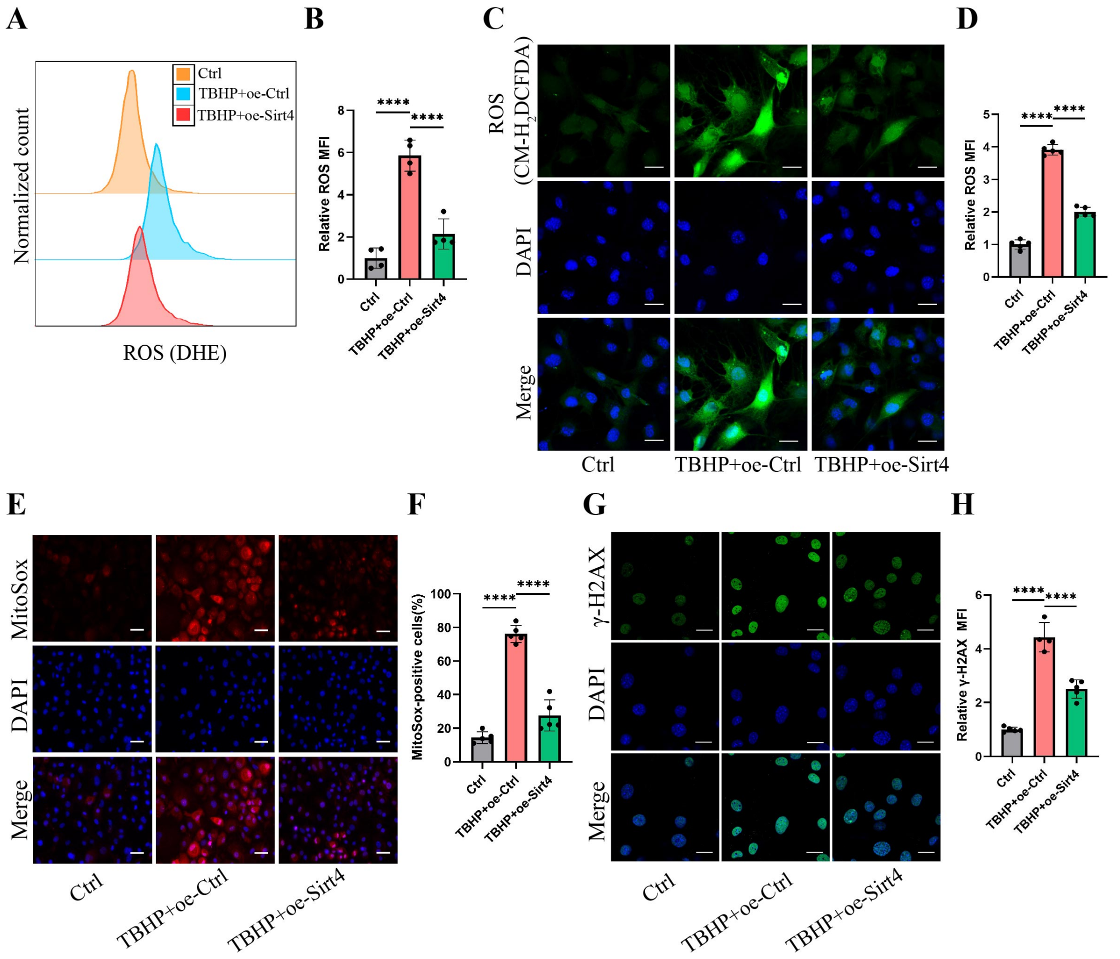

Supplementary figure 4. Sirt4 overexpression decreases TBHP-induced ROS production in chondrocytes. (A, B) Flow cytometer analysis of DHE fluorescence measuring levels of intracellular $O2^{\bullet-}$ in primary mouse chondrocytes transfected with oe-Sirt4 or oe-Ctrl and treated or untreated with TBHP (50 μM). The bar graph shows the fluorescence intensity of the DHE. n=4, ****p < 0.0001. (C, D) Confocal imaging analysis of CM-H_2DCFDA fluorescence measuring levels of intracellular ROS in primary mouse chondrocytes transfected with oe-Sirt4 or oe-Ctrl and treated or untreated with TBHP (50 μM). The bar graph shows the fluorescence intensity of the CM-H2DCFDA. n = 5, ****p < 0.0001, Scale bars: 20 μm. (E, F) Confocal imaging analysis of MitoSox fluorescence measuring levels of mitochondrial ROS in primary mouse chondrocytes transfected with oe-Sirt4 or oe-Ctrl and treated or untreated with TBHP (50 μM). The bar graph shows the fluorescence intensity of the MitoSox. n = 5, ****p < 0.0001, Scale bars: 20 μm. (G, H) Immunofluorescence staining of γ-H2AX and fluorescence intensity of γ-H2AX in primary mouse chondrocytes transfected with oe-Sirt4 or oe-Ctrl and treated or untreated with TBHP (50 μM). n = 5, ****p < 0.0001, Scale bars: 20 μm.

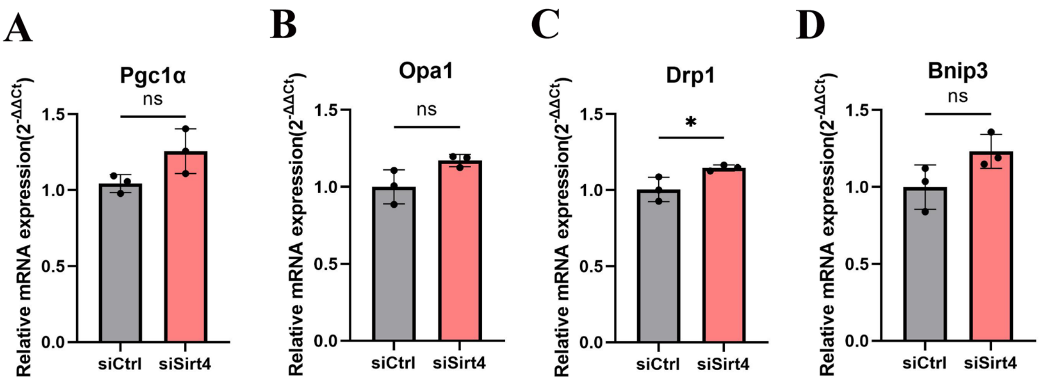

Supplementary figure 5. The mRNA expression level of genes relative to mitochondrial quality control after Sirt4 knockdown in chondrocytes. (A, B) RT-qPCR analysis of the expression level of Pgc1α, Opa1, Drp1 and Bnip3 in primary mouse chondrocytes transfected with siSirt4 or siCtrl. β-actin was used as normalization control. n = 3. *p < 0.05, **p < 0.01.

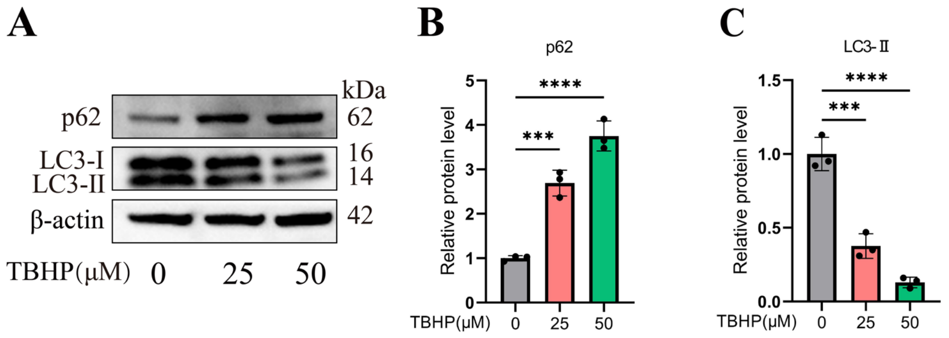

Supplementary Figure 6. Autophagy is inhibited in TBHP-induced cellular senescence model in chondrocytes. (A-C) Western blotting analysis of p62 and LC3-II protein levels in primary mouse chondrocytes treated with various concentrations of TBHP for 48h. Band intensity relative to β-actin. n=3, ***p < 0.001, ****p < 0.0001.

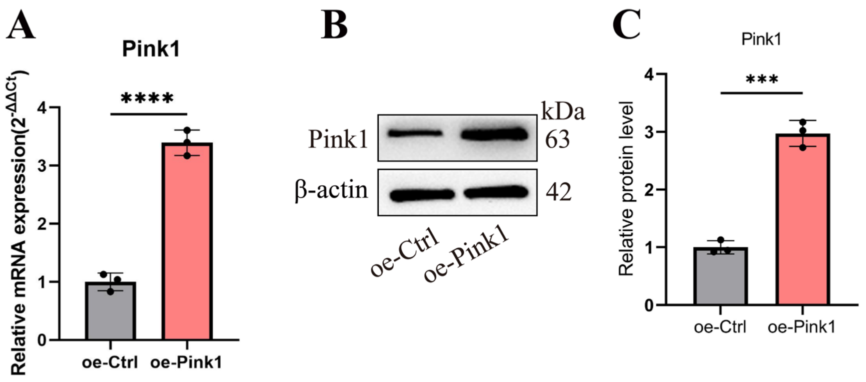

Supplementary figure 7. Transfection efficiency of plasmid encoding Pink1 in primary mouse chondrocytes. (A) Relative mRNA expression level of Pink1 in primary mouse chondrocytes transfected with plasmid encoding Pink1 (oe-Pink1) compared to plasmid control with no insert (oe-Ctrl). β-actin was used as normalization control. n = 3, ****$p < 0.0001$. (B, C) Western blotting analysis of Pink1 protein level in primary mouse chondrocytes transfected with oe-Pink1 plasmid compared to oe-Ctrl. Band intensity relative to β-actin. n = 3, ***$p < 0.001$.

Supplementary Tables

Supplementary Table 1. List of primers used in the present study.

Primers for qPCR		
Genes	Primer sequences(5′-3′)	
Sirt4	F	GTGGAAGAATAAGAATGAGCGGA
	R	GGCACAAATAACCCCGAGG
Mmp13	F	CTTCTTCTTGTTGAGCTGGACTC
	R	CTGTGGAGGTCACTGTAGACT
Col2a1	F	TGAAGACCCAGACTGCCTCAA
	R	AGCCGCGAAGTTCTTTTCTCC
p16	F	CGCAGGTTCTTGGTCACTGT
	R	TGTTCACGAAAGCCAGAGCG
p21	F	CCTGGTGATGTCCGACCTG
	R	CCATGAGCGCATCGCAATC
Cxcl10	F	CCAAGTGCTGCCGTCATTTTC
	R	GGCTCGCAGGGATGATTTCAA
IL-6	F	TAGTCCTTCCTACCCCAATTTCC
	R	TTGGTCCTTAGCCACTCCTTC
Mcp1	F	TTAAAAACCTGGATCGGAACCAA
	R	GCATTAGCTTCAGATTTACGGGT
Mmp3	F	ACATGGAGACTTTGTCCCTTTTG
	R	TTGGCTGAGTGGTAGAGTCCC
IL-1β	F	GCAACTGTTCCTGAACTCAACT
	R	ATCTTTTGGGGTCCGTCAACT
TNFα	F	CCCTCACACTCAGATCATCTTCT

	R	GCTACGACGTGGGCTACAG
IL-17	F	TTTAACTCCCTTGGCGCAAAA
	R	CTTTCCCTCCGCATTGACAC
Mmp9	F	CTGGACAGCCAGACACTAAAG
	R	CTCGCGGCAAGTCTTCAGAG
Pink1	F	TTCTTCCGCCAGTCGGTAG
	R	CTGCTTCTCCTCGATCAGCC
p62	F	ATGTGGAACATGGAGGGAAGA
	R	GGAGTTCACCTGTAGATGGGT
Pgc1α	F	TATGGAGTGACATAGAGTGTGCT
	R	CCACTTCAATCCACCCAGAAAG
Opa1	F	TGGAAAATGGTTCGAGAGTCAG
	R	CATTCCGTCTCTAGGTTAAAGCG
Mfn1	F	CCTACTGCTCCTTCTAACCCA
	R	AGGGACGCCAATCCTGTGA
Drp1	F	TTACGGTTCCCTAAACTTCACG
	R	GTCACGGGCAACCTTTTACGA
Bnip3	F	TCCTGGGTAGAACTGCACTTC
	R	GCTGGGCATCCAACAGTATTT
Parkin	F	TCTTCCAGTGTAACCACCGTC
	R	GGCAGGGAGTAGCCAAGTT
Cat	F	AGCGACCAGATGAAGCAGTG
	R	TCCGCTCTCTGTCAAAGTGTG
Gpx1	F	AGTCCACCGTGTATGCCTTCT
	R	GAGACGCGACATTCTCAATGA
Sod2	F	CAGACCTGCCTTACGACTATGG
	R	GCAGGATGGTAGTATGATT
Nfe2l2	F	TCTTGGAGTAAGTCGAGAAGTGT
	R	GTTGAAACTGAGCGAAAAAGGC
β-actin	F	CTCTGGCTCCTAGCACCATGAAGA
	R	GTAAAACGCAGCTCAGTAACAGTCCG

Supplementary Table 2. siRNAs used in the current study.

siRNAs	Dose	Sequence of validated siRNAs
Negative control	100 nM	Sense siRNA targeted sequence 5-CCUACGCCACCAAUUUCGU-3′<br>Antisense siRNA targeted sequence 5-ACGAAAUUGGUGGCGUAGG-3′
siSirt4#1	100 nM	Sense siRNA targeted sequence 5-UGGAGAGUUGCUGCCUUUAAU-3′<br>Sense siRNA targeted sequence 5-AUUAAAGGCAGCAACUCUCCA-3′
siSirt4#2	100 nM	Sense siRNA targeted sequence 5-AGUAAACCACAACUGUCUAUG-3′<br>Sense siRNA targeted sequence 5-CAUAGACAGUUGUGGUUUACU-3′

Supplementary Table 3 Synovitis score*

**Parameter**	**Grade**	**Description**
Synovial membrane inflammation	0	No changes (1–2 layers of synovial lining cells)
	1	Increased number of lining cell layers (≥3–4 layers) or slight proliferation of sub-synovial tissue.
	2	Increased number of lining cell layers (≥3–4 layers) and/or proliferation of sub-synovial tissue.
	3	Increased number of lining cell layers (>4 layers) and/or proliferation of sub-synovial tissue and infiltration of few inflammatory cells.
	4	Increased number of lining cell layers (>4 layers) and/or proliferation of sub-synovial tissue, infiltration of large number of inflammatory cells.

* Modified based on the scoring system described before [1].

1.Gerwin N, Bendele AM, Glasson S, Carlson CS. The OARSI histopathology initiative–recommendations for histological assessments of osteoarthritis in the rat. Osteoarthritis Cartilage. 2010;18:S24-S34.
